# Oxidative cleavage of cellulose in the horse gut

**DOI:** 10.1186/s12934-022-01767-8

**Published:** 2022-03-12

**Authors:** Ning Liu, Weishuai Yu, Xiuna Guo, Jinyin Chen, Donghui Xia, Jie Yu, Duochuan Li

**Affiliations:** Department of Mycology, Shandong Agricultural University, Taian, Shandong 271018 China

**Keywords:** Lytic polysaccharide monooxygenase (LPMO), Horse gut, C1 and C4 oxidation, Thermophilic fungi

## Abstract

**Background:**

Lytic polysaccharide monooxygenases (LPMOs) belonging to the auxiliary activity 9 family (AA9) are widely found in aerobic fungi. These enzymes are O_2_-dependent copper oxidoreductases that catalyze the oxidative cleavage of cellulose. However, studies that have investigated AA9 LPMOs of aerobic fungi in the herbivore gut are scare. To date, whether oxidative cleavage of cellulose occurs in the herbivore gut is unknown.

**Results:**

We report for the first time experimental evidence that AA9 LPMOs from aerobic thermophilic fungi catalyze the oxidative cleavage of cellulose present in the horse gut to C1-oxidized cellulose and C1- and C4-oxidized cello-oligosaccharides. We isolated and identified three thermophilic fungi and measured their growth and AA9 LPMO expression at 37 °C in vitro. We also assessed the expression and the presence of AA9 LPMOs from thermophilic fungi in situ. Finally, we used two recombinant AA9 LPMOs and a native AA9 LPMO from thermophilic fungi to cleave cellulose to yield C1-oxidized products at 37 °C in vitro.

**Conclusions:**

The oxidative cleavage of cellulose occurs in the horse gut. This finding will broaden the known the biological functions of the ubiquitous LPMOs and aid in determining biological significance of aerobic thermophilic fungi.

**Supplementary Information:**

The online version contains supplementary material available at 10.1186/s12934-022-01767-8.

## Background

Cellulose is a complex carbohydrate that consists of 3000 or more glucose residues. This polysaccharide is the structural component of plant cell walls and the most abundant of all naturally occurring organic compounds. Cellulose is not digestible by humans but is food for herbivores, such as cows and horses. These animals retain cellulose in their digestive systems long enough to be degraded by intestinal microorganisms. Gut microorganisms, also known as microbiota, include bacteria, archaea, and eukaryotes. Anaerobic fungi, an unusual group of zoosporic fungi common in the herbivore gut, produce enzymes, such as cellulases, for breaking down cellulose [1]. Known anaerobic fungi found in at least 50 different herbivore species, including horses, are classified as *Neocallimasticaceae.* This family includes 9 genera and over 29 species worldwide [2]. Complete genomes and transcriptomes of representative anaerobic fungi reveal an array of cellulose-degrading hydrolytic enzymes in different glucosyl hydrolase (GH) families, such as GH1, GH3, GH5, GH6, GH8, GH9, GH16, GH31, GH45, and GH48 [3–8]. However, anaerobic fungi seem to lack auxiliary activity 9 (AA9) enzymes, such as the recently discovered lytic polysaccharide monooxygenases (LPMOs) [3, 4, 6, 8].

AA9 LPMOs are common in aerobic fungi [9–11]. Genomic sequencing shows several AA9 LPMO genes in thermophilic fungi [9–12]. Publicly available genome annotations from such organisms indicate at least 24, 20, 4, 4, 6, and 18 AA9 genes predicted in *Thermothielavioides terrestris* (*Thielavia terrestris*), *Thermothelomyces thermophilus* (*Myceliophthora thermophila*), *Thermomyces lanuginosus*, *Thermoascus aurantiacus*, *Scytalidium thermophilum* (*Humicola insolens*), and *Chaetomium thermophilum*, respectively (www.fungalgenomics.ca, www.CAZy.org, ct.bork.embl.de/). AA9 LPMOs are O_2_-dependent copper oxidoreductases that catalyze the oxidative cleavage of cellulose, especially crystalline cellulose [11, 13]. LPMOs activate O_2_ to hydroxylate cellulose leading to the elimination of the glycosidic bond. The elimination reaction occurs upon hydroxylation of the C1 and C4 carbons. Recently, LPMOs have been shown to also use hydrogen peroxide (H_2_O_2_) as an oxidant for rapidly driving the elimination reaction [13]. AA9 LPMOs have been studied intensively, with a focus on their reaction mechanism, substrate specificity, 3-D structure, regioselectivity, activity assay, action mode, synergy with cellulase, and thermostability [11, 13–19]. However, few studies have investigated AA9 LPMOs of aerobic fungi in the herbivore gut. To date, oxidative cleavage of cellulose in herbivore gastrointestinal (GI) tracts has not been demonstrated.

The horse is an herbivorous mammal of the family Equidae. Horses have a relatively long digestive tract that harbors up to 10^8^ microorganisms/g [20]. The enzymatic machinery encoded by microorganisms is the sole contributor to the degradation of cellulose [21]. Recently, microbiomes of the horse gut have provided an increasingly comprehensive understanding of the cellulose cleavage in vivo [22–28]. Herein, we report experimental evidence that AA9 LPMOs from aerobic thermophilic fungi catalyze the oxidative cleavage of cellulose in the horse gut. We identified C1-oxidized cellulose and C1- and C4-oxidized cello-oligosaccharides in the horse gut. We isolated and identified three thermophilic fungi from the horse gut and measured their growth and AA9 LPMO expression in vitro at 37 °C, which is the typical body temperature of a horse. We also identified the expression and the presence of AA9 LPMOs from thermophilic fungi in the equine digestive system. Finally, we heterologously expressed two AA9 LPMOs and isolated a native AA9 LPMO of thermophilic fungi, which could oxidatively cleave cellulose at 37 °C.

## Results

### Identification of C1-oxidized cellulose and C1- and C4-oxidized cello-oligosaccharides in the horse gut

**Fig. 1:**
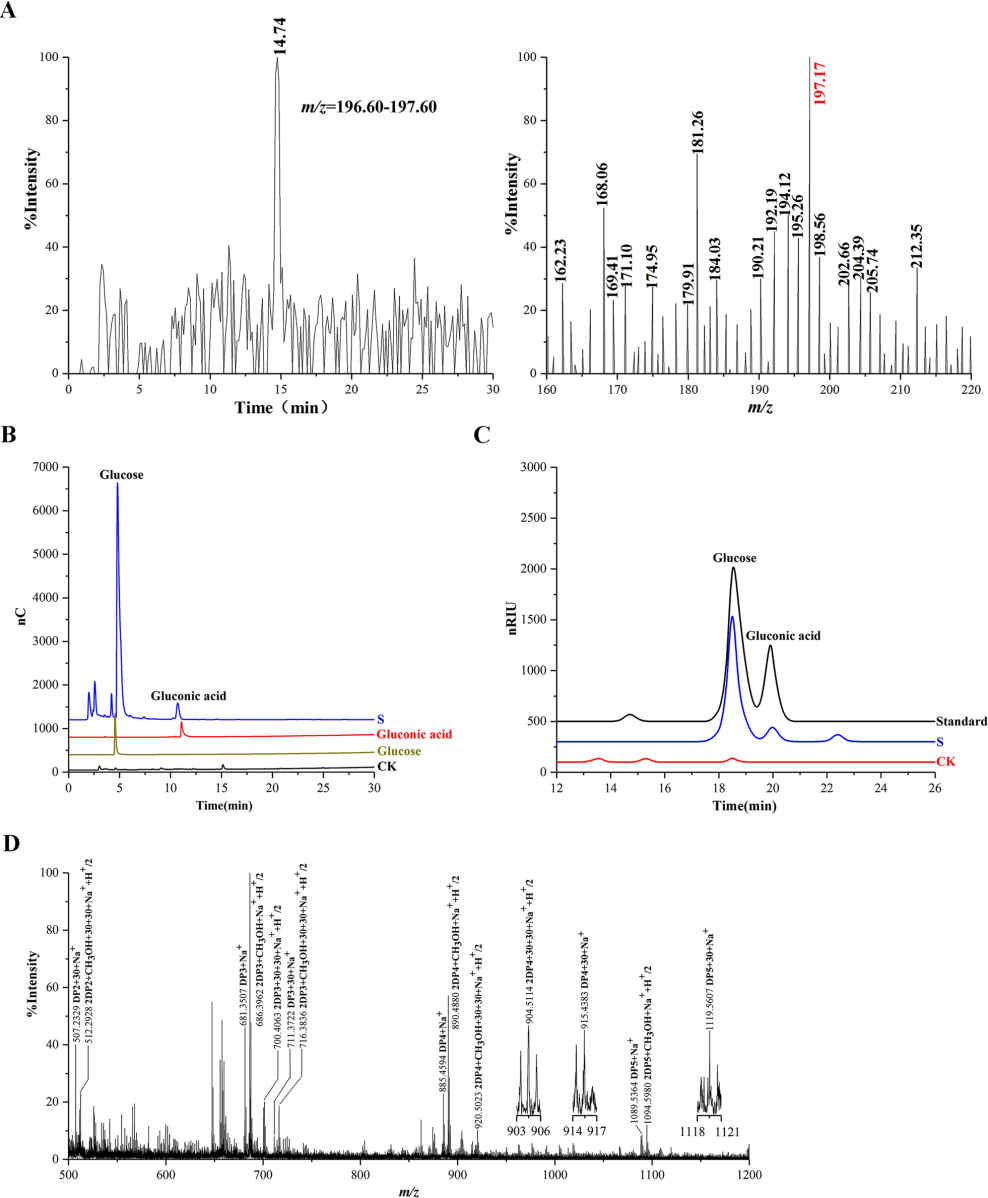
Identification of C1-oxidized products of the digested cellulose isolated from fresh horse feces. **A** LC-MS: The digested cellulose hydrolyzed by endocellulase followed by hydrolysis with TFA. LC-MS showing extraction chromatograms and corresponding mass spectra of gluconic acid. In positive mode: gluconic acid (*m/z* 196 + H^+^). **B** HPAEC-PAD: The digested cellulose hydrolyzed by endocellulase followed by hydrolysis with TFA. S, the digested cellulose; CK, the control sample analyzed as above except without the digested celllulose; Standards, glucose, and gluconic acid. **C** HPLC-RID: The digested cellulose was hydrolyzed by endocellulase followed by hydrolysis with TFA. C1-oxidized products are hydrolyzed by TFA to glucose and gluconic acid. Standard, a mixture of glucose and gluconic acid; S: the digested cellulose; CK, the control sample analyzed as above except without the digested cellulose. **D** MALDI-TOF-MS: The digested cellulose hydrolyzed by endocellulase followed by permethylation with methyl iodide. C1-oxidized cello-oligosaccharides (*m/z* + 30), non-oxidized cello-oligosaccharides (*m/z* + 0). Endocellulase reaction products permethylated by methyl iodide were dissolved in methanol, and molecular ion peaks of CH_3_OH occur in MALDI-TOF-MS spectra

**Fig. 2:**
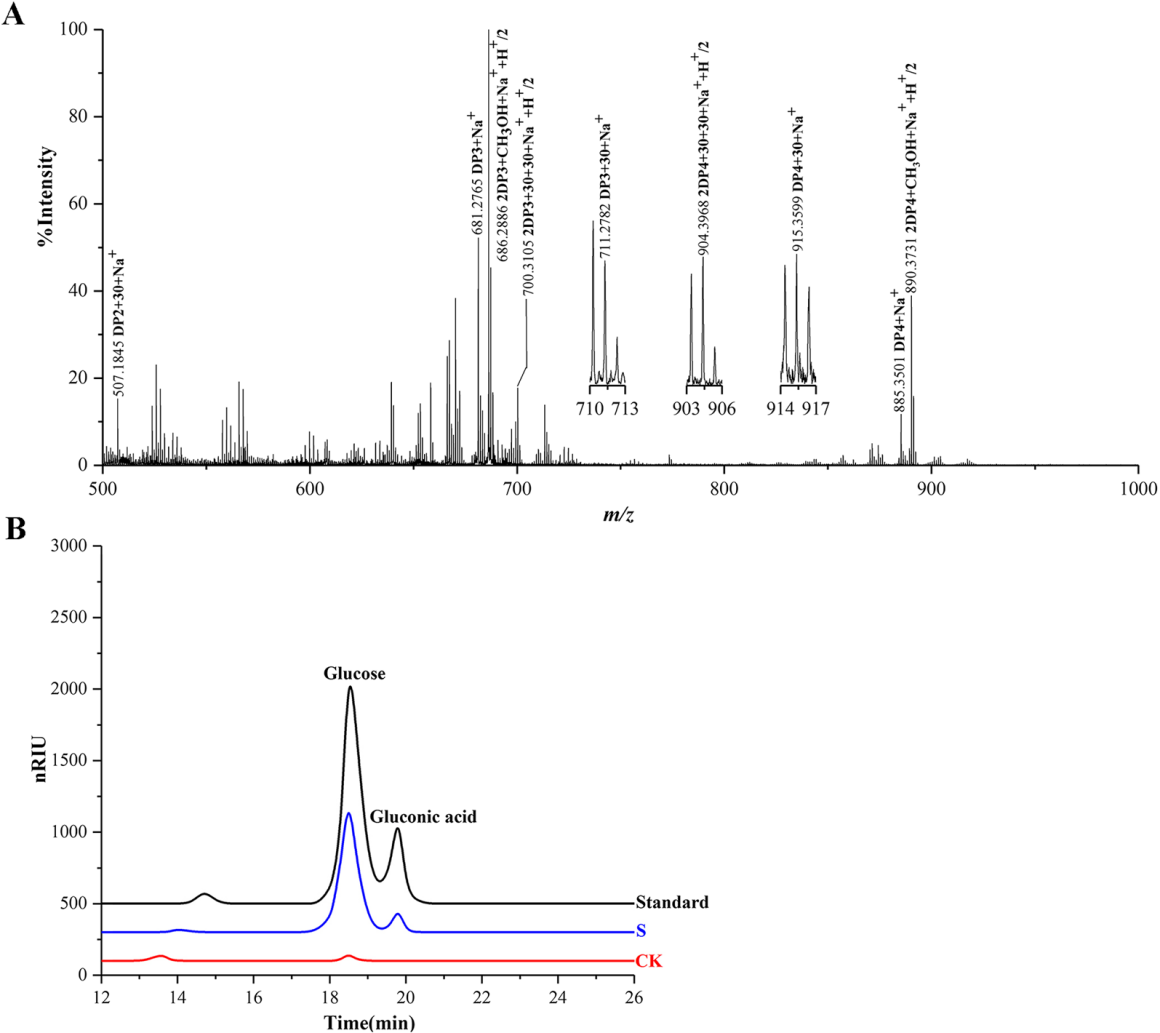
Identification of C1-oxidized products in the digested cellulose isolated from horse stomach using MALDI-TOF-MS and HPLC-RID. **A** MALDI-TOF-MS: The digested cellulose was hydrolyzed by endocellulase followed by permethylation with methyl iodide. C1-oxidized cello-oligosaccharides (*m/z* + 30), non-oxidized cello-oligosaccharides (*m/z* + 0). Some molecular ion peaks with CH_3_OH occur in MALDI-TOF-MS spectra. **B** HPLC-RID: The digested cellulose was hydrolyzed by endocellulase followed by hydrolysis with TFA. C1-oxidized products are hydrolyzed by TFA to glucose and gluconic acid. Standard, a mixture of glucose and gluconic acid; S: The digested cellulose; CK, the control sample analyzed as above except without the digested celllulose

**Fig. 3:**
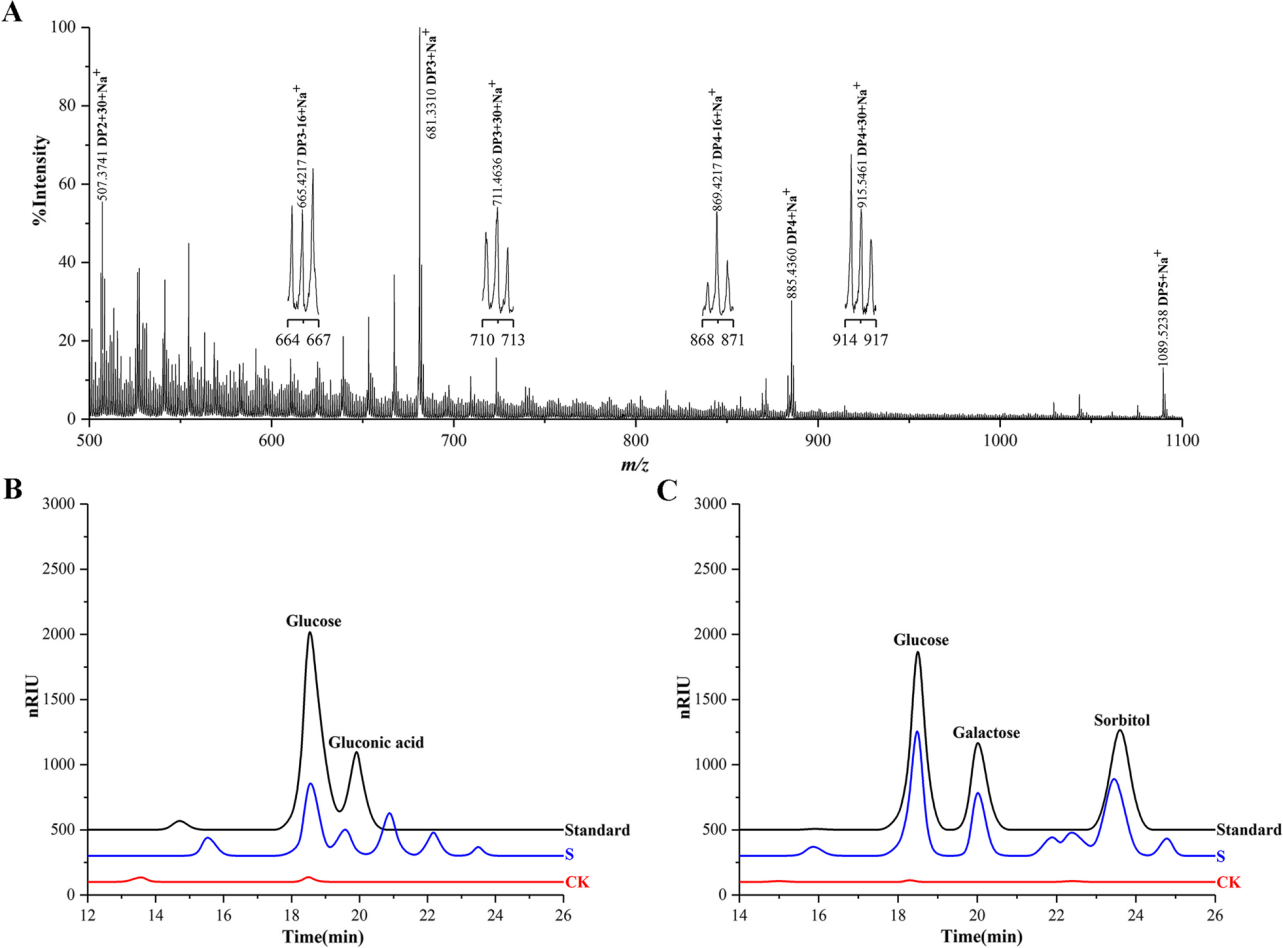
Identification of C1- and C4-oxidized products in the soluble oligosaccharides extracted from the horse stomach using MALDI-TOF-MS and HPLC-RID. **A** MALDI-TOF-MS: The extracted soluble oligosaccharides were permethylated with methyl iodide. C1-oxidized oligosaccharides (*m/z* + 30), C4-oxidized oligosaccharides (*m/z* − 16), non-oxidized oligosaccharides (*m/z* + 0). **B** HPLC-RID of C1-oxidized products: C1-oxidized products are hydrolyzed by TFA to glucose and gluconic acid. S: the soluble oligosaccharides; CK, control sample analyzed as above except without the soluble oligosaccharides; Standard, a mixture of glucose and gluconic acid. **C** HPLC-RID of C4-oxidized products: C4-oxidized products soluble cello-oligosaccharides are reduced by NaBH_4_ followed by hydrolysis with TFA to yield glucose, galactose, and sorbitol. Standard, a mixture of glucose, galactose, and sorbitol; S: the soluble oligosaccharides; CK, the control sample analyzed as above except without the soluble oligosaccharides

We isolated insoluble digested cellulose from fresh horse feces to assess the presence of C1- and C4-oxidized cellulose. We hydrolyzed the digested cellulose with an endoglucanase from *Acidothermus cellulolyticus* (AcEG) to yield soluble reaction products, and further hydrolyzed these reaction products with trifluoroacetic acid (TFA) to yield monosaccharides. We observed the C1-oxidized monosaccharide, gluconic acid (*m/z* 196 + H^+^), in hydrolysis products using liquid chromatography-mass spectrometry (LC-MS) (Fig. 1A). The presence of gluconic acid was also shown using high-performance anion exchange chromatography with pulsed amperometric detection (HPAEC-PAD) and high-performance liquid chromatography-refractive index detector (HPLC-RID) analysis (Fig. 1B and C). It should be pointed out that the retention time of peaks representing glucose and gluconic acid had a minor difference with that of glucose and gluconic acid standards in HPAEC-PAD analysis. The minor difference may be caused by the high concentration of glucose. Furthermore, we confirmed the presence of C1-oxidized products using a previously described chemical method by utilizing methyl iodide to permethylate AcEG reaction products [29]. As expected, we observed a series of molecular ions corresponding to C1-oxidized and non-oxidized cello-oligosaccharides using matrix-assisted laser desorption/ionization-time-of-flight mass spectrometry (MALDI-TOF-MS) (Fig. 1D, Additional file 1: Fig. S1). Similarly, we observed C1-oxidized cellulose in the insoluble digested cellulose isolated from the horse stomach with MALDI-TOF-MS and HPLC-RID analysis (Fig. 2A and B). C1-oxidized cellulose thus exists in the horse GI tract. Subsequently, we extracted soluble rumen fluid from the horse stomach. Again, we observed C1- and C4-oxidized cello-oligosaccharides using MALDI-TOF-MS and HPLC-RID (Fig. 3A and B, Additional file 1: Fig. S1), indicating the existence of C1- and C4-oxidized cello-oligosaccharides in the horse GI tract.

### Isolation and identification of thermophilic fungi from the horse gut

**Fig. 4:**
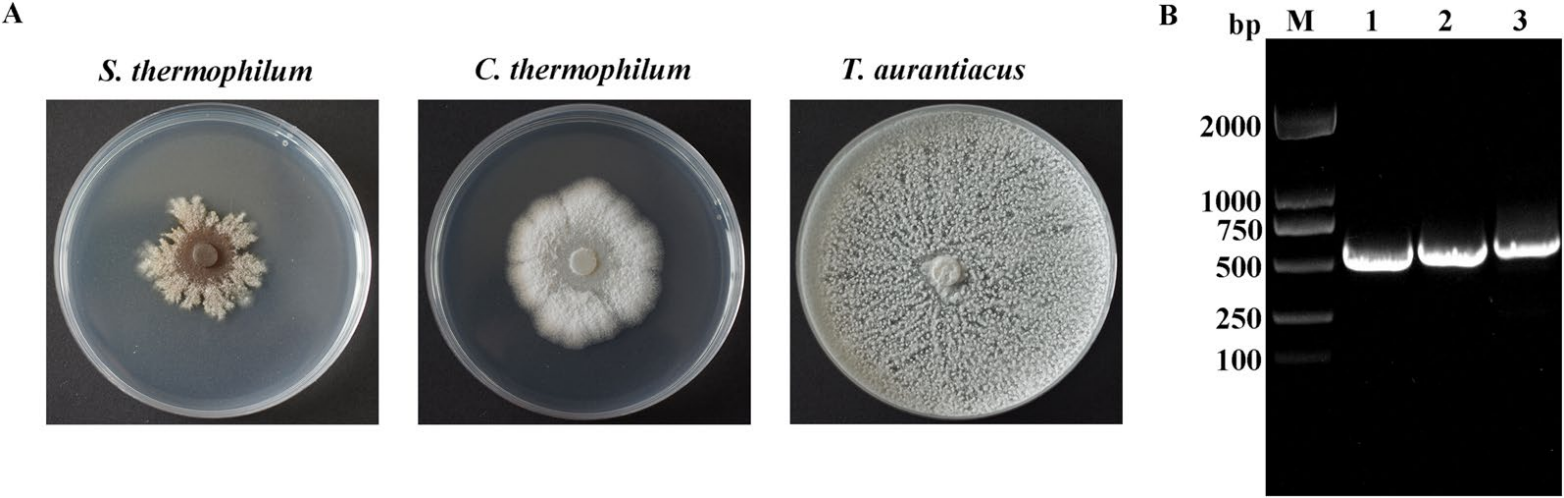
Isolation and identification of the thermophilic fungi in the fresh horse feces. **A** The growth of the three isolated thermophilic fungi, *S*. *thermophilum*, *C*. *thermophilum* and *T*. *aurantiacus*, on potato dextrose (PDA) agar medium incubated at 50 °C for 3 days. **B** Agarose gel electrophoresis of PCR-amplified ITS regions of *S*. *thermophilum*, *C*. *thermophilum* and *T*. *aurantiacus* using ITS1 and ITS4. Lane M, marker; Lane 1, *S*. *thermophilum*; Lane 2, *C*. *thermophilum*; Lane 3, *T*. a*urantiacus*

We isolated thermophilic fungi in the horse gut at a temperature of 50 °C, and identified three thermophilic species *Scytalidium thermophilum*, *Chaetomium thermophilum*, and *Thermoascus aurantiacus*, from fresh horse feces (Fig. 4A and B, Additional file 1: Tables S1 and S2), in accordance with our previous reports describing thermophilic fungi isolated from fresh horse feces [30, 31]. Thermophilic fungi are present in the horse gut microbiota. Similarly, aerobic *Ascomycota* fungi are reported as dominant members in fresh horse feces [32].

### Growth of thermophilic fungi and AA9 LPMO expression at 37 °C on cellulose-containing medium

**Fig. 5:**
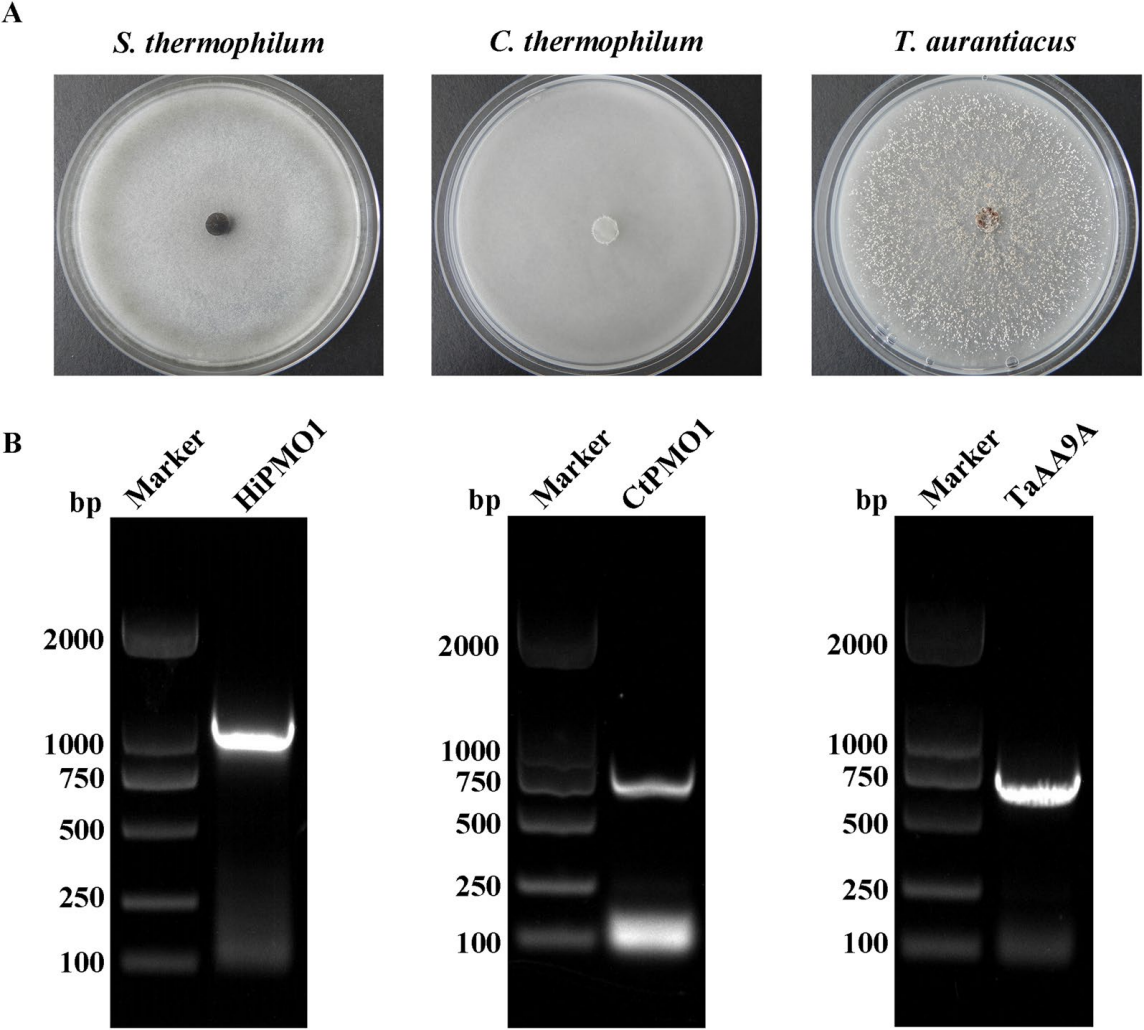
The growth and AA9 LPMO expression of the three thermophilic fungi on cellulose-containing medium at 37 °C. **A** The three isolated thermophilic fungi were cultured on cellulose-containing agar medium at 37 °C for 7 days. **B** Expression of AA9 LPMOs from the three isolated thermophilic fungi at 37 °C on cellulose-containing medium for 7 days using RT-PCR. Selected AA9 LPMOs are HiPMO1 from *S*. *thermophilum*, CtPMO1 from *C*. *thermophilum*, and TaAA9A from *T*. *aurantiacus*. The amplified PCR products were sequenced and confirmed to be HiPMO1, CtPMO1, and TaAA9A

The typical horse body temperature is 37 °C. We measured the growth of three isolated thermophilic fungi *S*. *thermophilum*, *C*. *thermophilum* and *T*. *aurantiacus*, and their AA9 LPMO expression at this temperature on cellulose-containing medium in vitro. As expected, the fungi grew well at 37 °C on cellulose-containing media (Fig. 5A). In addition, three AA9 LPMOs, CtPMO1 from *C*. *thermophilum* [29], HiPMO1 from *S*. *thermophilum* [33], and TaAA9A from *T*. *aurantiacus* [34, 35], were expressed at 37 °C on cellulose-containing media (Fig. 5B). These enzymes were similarly expressed at 50 °C (Additional file 1: Fig. S2A and B) [29, 33, 35]. Thus, thermophilic fungi are expected to grow and AA9 LPMOs are expected to be expressed in the equine GI tract.

### Expression and identification of thermophilic fungal LPMOs in the horse gut

**Fig. 6:**
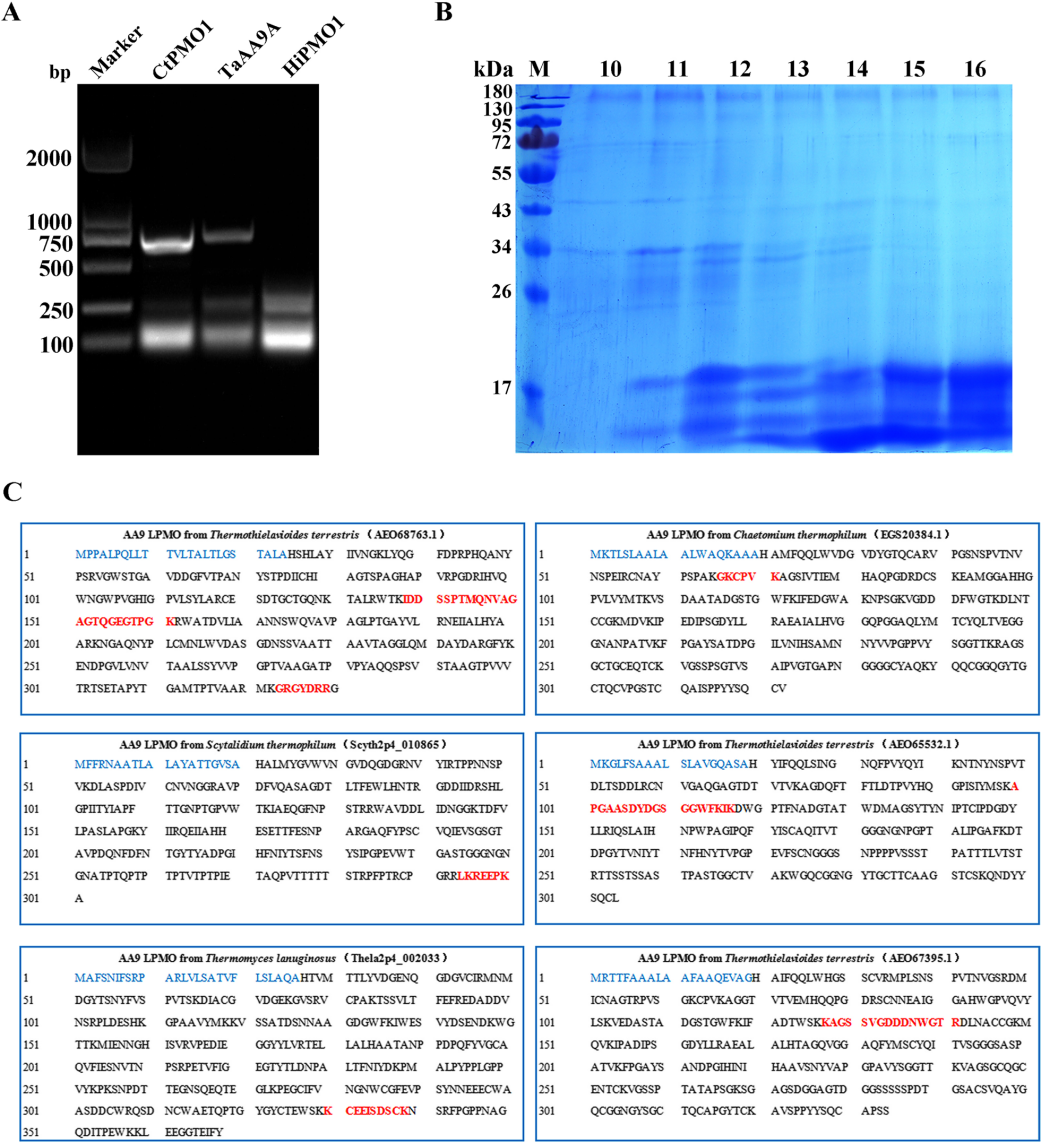
Expression and identification of AA9 LPMOs of thermophilic fungi in the horse gut. **A** Expression of AA9 LPMOs in the horse stomach using RT-PCR: Selected AA9 LPMOs are CtPMO1, TaAA9A, and HiPMO1. Amplified PCR products were sequenced and confirmed to be CtPMO1 and TaAA9A but HiPMO1 was not observed. **B** Identification of AA9 LPMOs of thermophilic fungi using SDS-PAGE: Proteins from the horse gut were isolated by ion-exchange chromatography on a DEAE-sepharose column. Isolated proteins were visualized by staining with Coomassie Brilliant Blue. M, protein marker (170, 130, 95, 72, 55, 43, 34, 26 kDa); Fractions of proteins from DEAE-sepharose column were shown in 10, 11, 12, 13, 14, 15, and 16, respectively. **C** Identification of AA9 LPMOs of thermophilic fungi using LC-MS/MS: Isolated proteins were digested with trypsin. The resulting peptides matched to AA9 LPMOs from thermophilic fungi were shown in red. The singal peptide sequences were shown in blue

Total RNA was isolated from the fresh digesta. We used reverse transcription-polymerase chain reaction (RT-PCR) to assess the expression of two AA9 LPMOs (CtPMO1 from *C*. *thermophilum* and TaAA9A from *T*. *aurantiacus*) in the horse stomach (Fig. 6A). We then isolated thermophilic fungal AA9 LPMOs from the horse stomach using ion-exchange chromatography on DEAE-sepharose column and identified the enzymes using sodium dodecyl sulfate-polyacrylamide gel electrophoresis (SDS-PAGE) and liquid chromatography-tandem mass spectrometry (LC-MS/MS). We observed six AA9 LMPOs of thermophilic fungi, three from *T*. *terrestris*, one from *C*. *thermophilum*, one from *T. lanuginosus*, and one from *S*. *thermophilum* (Fig. 6B and C, Additional file 1: Fig. S3, Additional file 2: Table S1 and S2). Thermophilic fungi normally express AA9 LPMOs and also secrete them effectively to cleave cellulose in the horse GI tract.

### Identification of C1-oxidized cello-oligosaccharides produced by AA9 LPMOs from the isolated thermophilic fungi at 37 °C

**Fig. 7:**
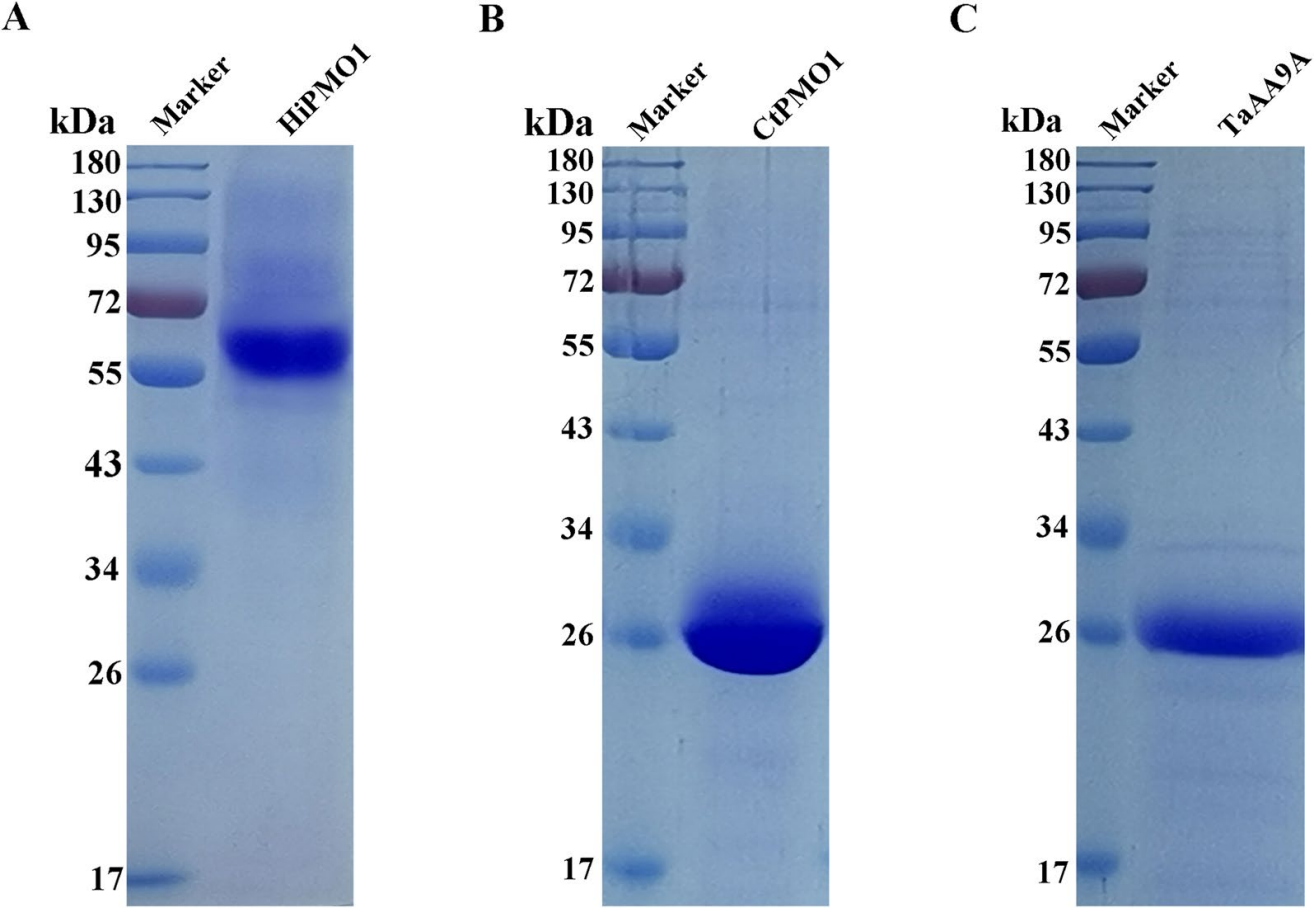
SDS-PAGE of the purified CtPMO1 and HiPMO1 and the isolated TaAA9A. CtPMO1, HiPMO1, and TaAA9A were visualized by staining with Coomassie Brilliant Blue. M, protein marker (170, 130, 95, 72, 55, 43, 34, 26, 17, 10 kDa). **A** HiPMO1 purified using nickel affinity chromatography. **B** CtPMO1 purified using nickel affinity chromatography. **C** TaAA9A isolated using ion-exchange chromatography on DEAE-sepharose column

**Fig. 8:**
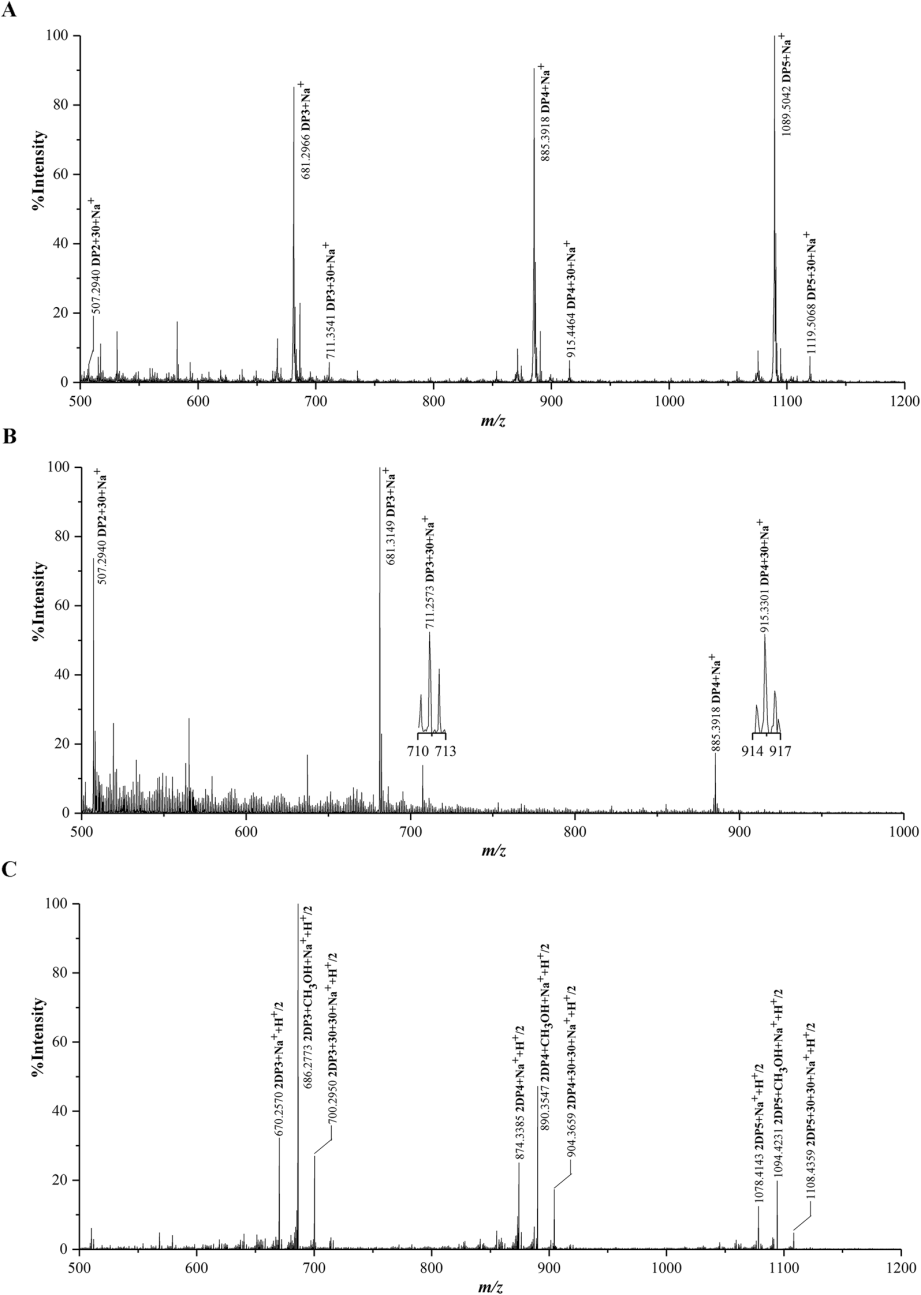
Identification of C1-oxidized cello-oligosaccharides catalyzed by AA9 LPMOs of thermophlic fungi in the horse gut at 37 ˚C using MALDI-TOF-MS. The two recombinant AA9 LPMOs (CtPMO1 and HiPMO1) and the native AA9 LPMO (TaAA9A) were used to cleave cellulose at 37 °C. C1-oxidized cello-oligosaccharides (*m/z* + 30), non-oxidized cello-oligosaccharides (*m/z* + 0). Some molecular ion peaks of CH_3_OH occur in MALDI-TOF-MS spectra of TaAA9A reaction products. **A** HiPMO1 reaction products. **B** CtPMO1 reaction products. **C** TaAA9A reaction products

**Fig. 9:**
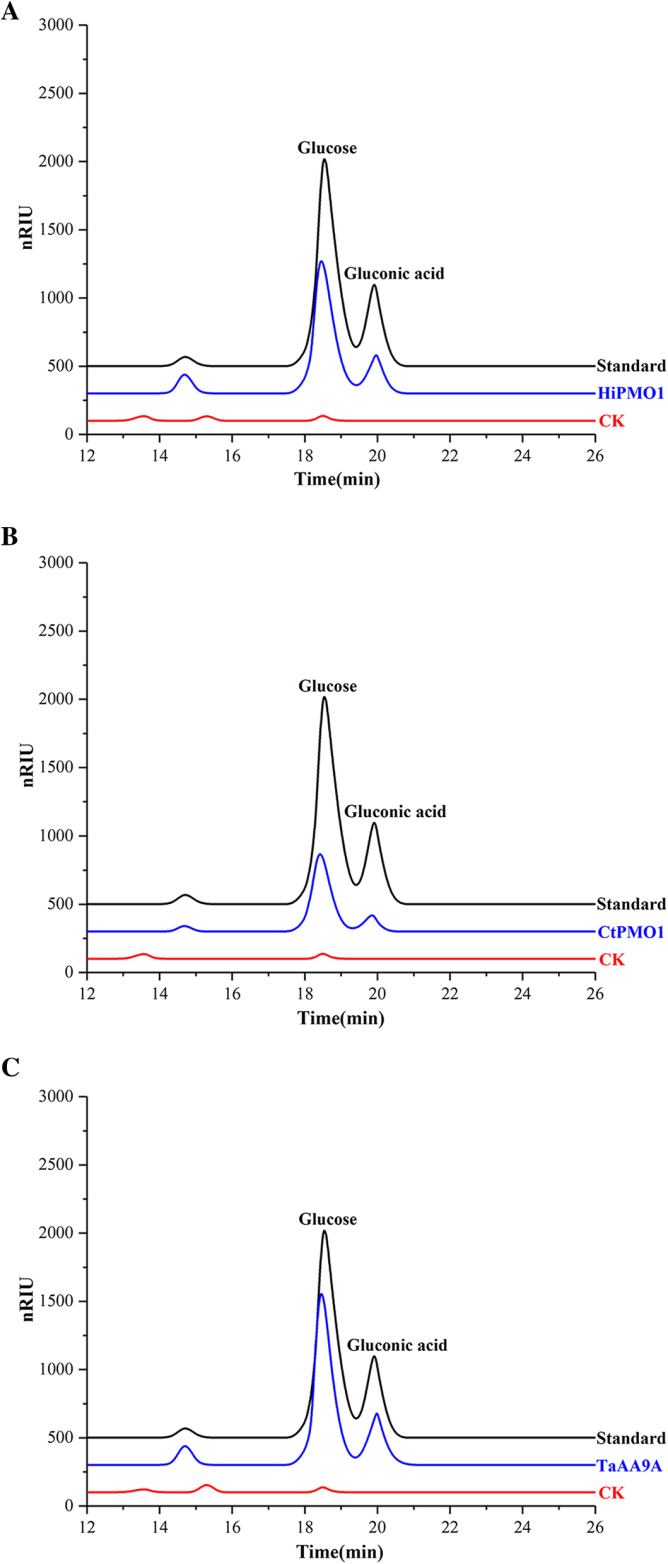
Identification of C1-oxidized cello-oligosaccharides catalyzed by AA9 LPMOs of thermophlic fungi in the horse gut at 37 ˚C using HPLC-RID. LPMO reaction products were hydrolyzed by TFA. C1-oxidized products are hydrolyzed by TFA to glucose and gluconic acid. Standard, a mixture of glucose and gluconic acid; S, LPMO reaction products; CK, the control sample analyzed as above except without LPMOs. **A** HiPMO1 reaction products. **B** CtPMO1 reaction products. **C** TaAA9A reaction products

Two recombinant AA9 LPMOs, HiPMO1 from *S*. *thermophilum* [33] and CtPMO1 from *C*. *thermophilum* [29], and one native AA9 LPMO of approximately 26 kDa, TaAA9A from *T*. *aurantiacus* [34, 35], were used to catalyze the cleavage of cellulose at 37 °C (Fig. 7A to C). We observed C1-oxidized cello-oligosaccharides in HiPMO1, CtPMO1 and TaAA9A reaction products using MALDI-TOF-MS (Fig. 8A to C). Further HPLC-RID analysis also showed the presence of C1-oxidized reaction products (Fig. 9A to C). These AA9 LPMOs can oxidatively cleave cellulose at 37 °C, further supporting the existence of the oxidative break down of cellulose in the horse gut.

## Discussion

The herbivore gut is a fascinating ecosystem where cellulose can be degraded by anaerobic fungi via enzymatic hydrolysis [1, 2, 5, 6]. We report for the first time oxidative cleavage of cellulose in the horse gut. AA9 LPMOs are found in many aerobic fungi, including thermophilic species [9–11], but not in anaerobic fungi [3, 4, 6, 8]. Therefore, we suggest that AA9 LPMOs from aerobic thermophilic fungi participate in the oxidative cleavage of cellulose in the horse gut.

Oxidative cleavage of cellulose is not a surprising phenomenon. Recently, aerobic fungal AA9 LPMOs were reported to be expressed to a considerable extent in the gut of wood-feeding termites [36]. This insect gut is aerobic and anaerobic from anterior to posterior, respectively [5, 37–39]. The expression of O_2_-dependent AA9 LPMOs in the termite gut is thus reasonable. The herbivore gut is also aerobic and anaerobic from anterior to posterior. Herbivores swallow air in the course of frequent daily feeding. In addition, O_2_ exists in herbivore saliva, which helps herbivorores chew and digest food. Horses can produce approximately 37 L of saliva per day. The existence of AA9 LPMOs, cellulose and O_2_ as well as a suitable reaction temperature are conducive for oxidative cleavage of cellulose in the horse gut.

Aerobic fungi produce multiple forms of AA9 LPMOs for cellulose degradation [9–12]. The thermophilic fungus *Thermothielavioides terrestris* produces at least six AA9 LPMOs [40]. In the present study, we observed the expression of CtPMO1 and TaAA9A but failed to detect HiPMO1. Furthermore, we identified only six LPMOs from aerobic thermophilic fungi. Two possible explanations are that (1) AA9 LPMOs may be spatially and temporally expressed and may exhibit differences in expression in response to the wide variety of plant biomass sources in the diet of horses [9, 12, 40, 41], and (2) some RNAs encoding AA9 LPMOs and AA9 LPMOs may be degraded or be present at low concentrations in the GI tract.

AA9 LPMOs cleave cellulose via C1 and C4 oxidation [11, 13]. We observed C1-oxidized cellulose in the digesta and C1- and C4-oxidized cello-oligosaccharides in the rumen fluid. C4-oxidized cellulose was not detected in the digesta as well as C4-oxidized cello-oligosaccharides in the reaction products of AA9 LPMOs. C4-oxidized cellulose in the digesta and C4-oxidized cello-oligosaccharides in AA9 LPMO reaction products may be present at concentrations that are too low to be detected. The lower dissociation energy of the C-O bond at C1 compared with C4 makes the former more easily broken, leading to a higher yield of C1-oxidized products. In addition, the lower reaction temperature (37 °C) may not be suitable for the formation of C4-oxidized products. Interestingly, AA9 LPMOs from thermophilic fungi produce C1 and C4 oxidation products from cellulose at a higher reaction temperature (50 °C) [29, 33, 34]. We postulate that the formation of such products may be a complex phenomenon in the horse gut and may be affected by factors, such as different AA9 LPMOs, temperature and oxygen concentration, all of which vary in the horse digestive system.

This novel finding of oxidative cleavage of cellulose in the horse gut broadens known biological functions of the ubiquitous AA9 LPMOs and illustrates the biological significance of aerobic thermophilic fungi. Aerobic fungi grow in the aerobic anterior region of the horse gut, where O_2_ is consumed and AA9 LPMOs are produced. These processes lead to oxidative cleavage of cellulose to yield cello-oligosaccharides. Consumption of O_2_ causes anaerobic conditions in the posterior gut where anaerobic fungi grow and produce cellulases. These enzymes hydrolyze cello-oligosaccharides to form glucose. This process accounts for the growth of aerobic fungi anteriorly that creates an anaerobic environment posteriorly. Moreover, AA9 LPMOs produced by aerobic fungi initially cleave crystalline cellulose to boost the enzymatic hydrolysis of cellulose by cellulases that are produced by anaerobic fungi in the posterior gut.

## Conclusions

We report for the first time the existence of oxidative cleavage of cellulose in the horse digesta. This finding broadens known biological functions of the ubiquitous AA9 LPMOs and illustrates the biological significance of aerobic thermophilic fungi in the horse digestive system.

## Materials and methods

### Plasmids, strains, and chemicals

The plasmid pPICZαA and *Pichia pastoris* GS115 were purchased from Invitrogen. Ascorbate, avicel PH-101, gluconic acid, galactose, sorbitol, and glucose were purchased from Sigma-Aldrich. Other chemicals were of analytical reagent grade and were obtained from Shandong Keshang Biotechnology (China).

### Sample collection

Fresh samples of horse feces and digested biomass from a horse stomach were collected from horse (Shandan horse and Mongolian horse) farms in Shandan County in Gansu Province, and Ningjin County in Shandong province, China. All fresh samples were frozen immediately in liquid nitrogen, transported on dry ice to the laboratory, and stored at − 86 °C before use.

### Isolation and identification of thermophilic fungi

We isolated thermophilic fungi from fresh horse feces as previously described [30, 31]. The isolated thermophilic fungi were identified by sequencing of the internal transcribed spacer (ITS) region. DNA was extracted from fungal mycelia from fresh cultures on potato dextrose (PDA) agar medium at 50 °C using a Fungal Genomic DNA extraction Kit (Solarbio, China). Fungal ITS regions were amplified using fungal-specific ITS1 and ITS4 primers (Additional file 1: Table S3). The PCR cycle was as follows: 94 °C for 3 min; 30 cycles of 94 °C for 40 s, 52 °C for 40 s, and 72 °C for 60 s; 72 °C for 10 min for final extension. Sequences were compared with available database sequences using BLAST at the National Center for Biotechnology Information (https://blast.ncbi.nlm.nih.gov). Sequences sharing ≥ 99% similarity were considered to represent identical species.

### Culture of thermophilic fungi

The isolated thermophilic fungi from the horse gut were grown in shake cultures at 37 °C in cellulose-containing medium [29]. After incubation for 7 days at 37 °C, mycelia were harvested by centrifugation at 5000 g for 5 min at 4 °C, frozen immediately in liquid nitrogen, and stored at − 86 °C for RT-PCR.

### Isolation and analysis of the digested cellulose from the horse gut

Fresh samples of horse feces and the digesta from the horse stomach were washed with distilled water to remove soluble material. The washed insoluble material was soaked in 10% NaOH for 8 h at room temperature and then at 100 °C for 3 h. After cooling, insoluble digested cellulose was separated by centrifugation at 5000 g for 10 min, washed with distilled water until the filtrate became clear, and dried under vacuum at room temperature. Dried material was hydrolyzed with an endo-1,4-beta-glucanase from *Acidothermus cellulolyticus* (Sigma-Aldrich) at 50 °C for 10 min at pH 5.0 (10 mM ammonium acetate), centrifuged at 10,000 g at 4 °C for 10 min, and the supernatant analyzed using LC-MS, HPAEC-PAD, HPLC-RID, and MALDI-TOF-MS.

### Extraction and analysis of soluble products

Soluble oligosaccharides were extracted from the fresh horse stomach digesta with distilled water. After 3 h at room temperature, the samples were centrifuged at 10,000 g for 15 min, and alcohol was added to the supernatant to a final concentration of 90%. After 12 h at room temperature, solid material was removed by centrifugation at 10,000**g** for 15 min, and the supernatant was concentrated in a vacuum evaporator at 30 °C for MALDI-TOF-MS and HPLC-RID analysis.

### Purification of recombinant CtPMO1 and HiPMO1 expressed in ***Pichia pastoris*** and isolation of the native TaAA9A from ***Thermoascus aurantiacus***

Recombinant CtPMO1 from *C*. *thermophilum* and HiPMO1 from *S*. *thermophilum* were expressed in *Pichia pastoris* and purified by nickel affinity chromatography as previously described [29, 33]. Native TaAA9A was isolated by ion-exchange chromatography on DEAE-sepharose column (GE Healthcare) from a 7-day culture filtrate of *T*. *aurantiacus* grown at 50 °C in cellulose-containing medium [29]. The isolated TaAA9A was visualized on an SDS-PAGE gel for confirmation as an AA9 LPMO (TaAA9A) [35].

### Isolation and identification of AA9 LPMOs from thermophilic fungi in the horse gut

Proteins from the horse stomach were extracted using radio immunoprecipitation assay (RIPA) lysis buffer (Beyotime, China). Extracted proteins were isolated using ion-exchange chromatography with a DEAE-Sepharose column (GE Healthcare). Fifty grams of fresh horse stomach sample was added to 100 ml of RIPA lysis buffer. The mixture was stirred for 3 h at 4 °C, and centrifuged at 10,000 g for 15 min at 4 °C, and the supernatant was dialyzed against 50 mM Tris-HCl (pH 8.0) (buffer A). Next, the dialyzed sample was placed on a DEAE-sepharose column equilibrated with buffer A. Proteins were eluted with 0.3 M NaCl in buffer A. Fractions with proteins were pooled and concentrated by vacuum freeze-drying at − 48 °C. Finally, the concentrated sample was analyzed by SDS-PAGE. Proteins in the concentrated sample were identified using LC-MS/MS. Briefly, the concentrated protein sample was desalted on an RP-C18 precolumn (Waters) and digested with trypsin (Sigma-Aldrich). Peptides were separated on a nano-ultra performance liquid chromatography (UPLC) RP-C18 column (Waters) of an Ultimate 3000 system coupled with a Q Exactive^™^ Hybrid Quadrupole-Orbitrap^™^ Spectrometer (Thermo Fisher Scientific) with an ESI nanospray source, working in concert with a data dependent MS to MS/MS switch with CID-type peptide fragmentation. Peptide masses and fragmentation spectra were matched to the database for AA9 LPMOs from thermophilic fungi (Additional file 1: Table S4) using MaxQuant (1.6.2.10). The parameters were set as follows: protein modifications were carbamidomethylation (C) (fixed), oxidation (M) (variable), acetyl (protein N-term) (variable); methyl (protein N-term) (variable). Enzyme specificity was normalized to trypsin, and the maximum missed cleavage was set to two; precursor ion mass tolerance was set to 20 ppm, and MS/MS tolerance was set to 20 ppm.

### Protein determination and SDS-PAGE

The Lowry method was used for total protein determination [42]. The purity of protein was determined using SDS-PAGE [43]. The gel system included a stacking gel (3% acrylamide) and a resolving gel (12% acrylamide). The protein was stained with 0.2% Coomassie brilliant blue R250.

### LPMO assay

Phosphoric acid swollen cellulose was prepared as described by Phillips et al. [44]. Activity assays were conducted as previously described [29, 33].

### MALDI-TOF-MS, LC-MS, HPAEC-PAD, and HPLC-RID

Endocellulase reaction products of the digesta, soluble products from the horse gut, and LPMO reaction products were identified using MALDI-TOF-MS, LC-MS, HPAEC-PAD, and HPLC-RID analyses. Products were permethylated by methyl iodide and dissolved in methanol for MALDI-TOF-MS analysis [29, 33]. The products were hydrolyzed with TFA for LC-MS analysis [35], and with TFA or reduced by NaBH_4_ followed by hydrolysis with TFA [45] for HPAEC-PAD and HPLC-RID analysis. HPAEC-PAD was conducted with a PA-200 HPAEC column as previously described, with a slight modification of flow rate to 0.3 ml/min [45]. HPLC-RID was conducted using an Agilent 1200 series instrument with an RID. Products were separated using an Aminex HPX-87 H column (Bio-Rad) and a 5 mM H_2_SO_4_ mobile phase. The flow rate was 0.3 ml/min, and the column was maintained at a temperature of 30 °C.

### RT-PCR

Isolated thermophilic fungi grown at 37 °C in cellulose-containing medium and fresh stomach samples were used for RNA isolation. Total RNA was extracted using an Ultrapure RNA Kit (CWBIO, China) and reverse-transcribed using TransScript All-in-One First-Strand cDNA Synthesis SuperMix for PCR systems (TransGen Biotech, China) following the manufacturer’s protocol, and cDNA samples were used for PCR amplification. The PCR cycle was: 94 °C for 3 min; 30 cycles of 94 °C for 40 s, 52 °C for 40 s and 72 °C for 60 s; and 72 °C for 10 min for final extension. PCR products were purified using a Gel Extraction Kit (Omega Bio-Tek) and sequenced by Sangon Biotech, China. The primers used are shown in Additional file 1: Table S3.

## Supplementary Information

**Additional file 1:** **Fig. S1. **The structure of LPMO reaction products. **Fig. S2. **Isolation of the native ***Thermoascus aurantiacus*** TaAA9A. **Fig. S3.** Identification of AA9 LPMOs of thermophilic fungi in the horse gut using LC-MS/MS. **Table S1.** ITS sequences of *Chaetomium thermophilum*, *Thermoascus aurantiacus*, *Scytalidium thermophilum*.  **Table S2.** Molecular identification of thermophilic fungi from fresh horse fecal using subunit 5.8 S rDNA gene (ITS1 and ITS4). **Table S3.** List of primers used for PCR in this study. **Table S4.** Data bank of protein sequences of AA9 LPMOs from thermophilic fungi.

**Additional file 2: Table S1.** Identification AA9 LPMOs of thermophilic fungi in the horse gut using LC-MS/MS. **Table S2.** Features of the peptides matched to AA9 LPMOs of thermophilic fungi in the horse gut in LC-MS/MS analysis.

## Data Availability

All data generated or analyzed during this study are included in this published article.

## Abbreviations

AA: Auxiliary activity
CtPMO1: An LPMO from *Chaetomium thermophilum*
HPAEC-PAD: High-performance anion exchange chromatography with pulsed amperometric detection
HPLC-RID: High-performance liquid chromatography-refractive index detector
HiPMO1: An LPMO from *Scytalidium thermophilum*
ITS: Internal transcribed spacer
LPMOs: Lytic polysaccharide monooxygenases
LC-MS: Liquid chromatography mass spectrometry
LC-MS/MS: Liquid chromatography-tandem mass spectrometry
TaAA9A: An LPMO from *Thermoascus aurantiacus*
MALDI-TOF-MS: Matrix-assisted laser desorption/ionization-time-of-flight mass spectrometry
PASC: Phosphoric acid-swollen cellulose
RT-PCR: Reverse transcription-polymerase chain reaction
RIPA: Radio immunoprecipitation assay
SDS-PAGE: Sodium dodecyl sulfate polyacrylamide gel electrophoresis
