# Supplementary material for: Oxidative cleavage of cellulose in the horse gut
Source: Microb Cell Fact. 2022 Mar 12;21:38. doi: 10.1186/s12934-022-01767-8 (PMC8917663; doi:10.1186/s12934-022-01767-8)
Supplement: Supplementary file 1 — Additional file 1: Fig. S1. The structure of LPMO reaction products. Fig. S2. Isolation of the native Thermoascus aurantiacus TaAA9A. Fig. S3. Identification of AA9 LPMOs of thermophilic fungi in the horse gut using LC-MS/MS. Table S1. ITS sequences of Chaetomium thermophilum, Thermoascus aurantiacus, Scytalidium thermophilum. Table S2. Molecular identification of thermophilic fungi from fresh horse fecal using subunit 5.8 S rDNA gene (ITS1 and ITS4). Table S3. List of primers used for PCR in this study. Table S4. Data bank of protein sequences of AA9 LPMOs from thermophilic fungi. [file 12934_2022_1767_MOESM1_ESM.docx]

**Additional file 1**

**Fig. S1. The structure of LPMO reaction products.** C1-oxidized oligosaccharides, C4-oxidized oligosaccharides, and non-oxidized oligosaccharides.

**

**

**Fig. S2. Isolation of the native *Thermoascus aurantiacus* TaAA9A.** (A) Chromatography of the native *Thermoascus aurantiacus* TaAA9A isolated by ion-exchange chromatography on DEAE-sepharose column. (B) SDS-PAGE of the native *Thermoascus aurantiacus* TaAA9A isolated by ion-exchange chromatography on DEAE-sepharose column. TaAA9A was visualized by staining with Coomassie Brilliant Blue. M, protein marker (170, 130, 95, 72, 55, 43, 34, 26, 17, 10 kDa); 6-13 corresonds to fractions of proteins from DEAE-sepharose column, respectively. TaAA9A visulised on a SDS-PAGE gel was confirmed to be an AA9 LPMO according to previously reported data from *Thermoascus aurantiacus* [35].

**A**


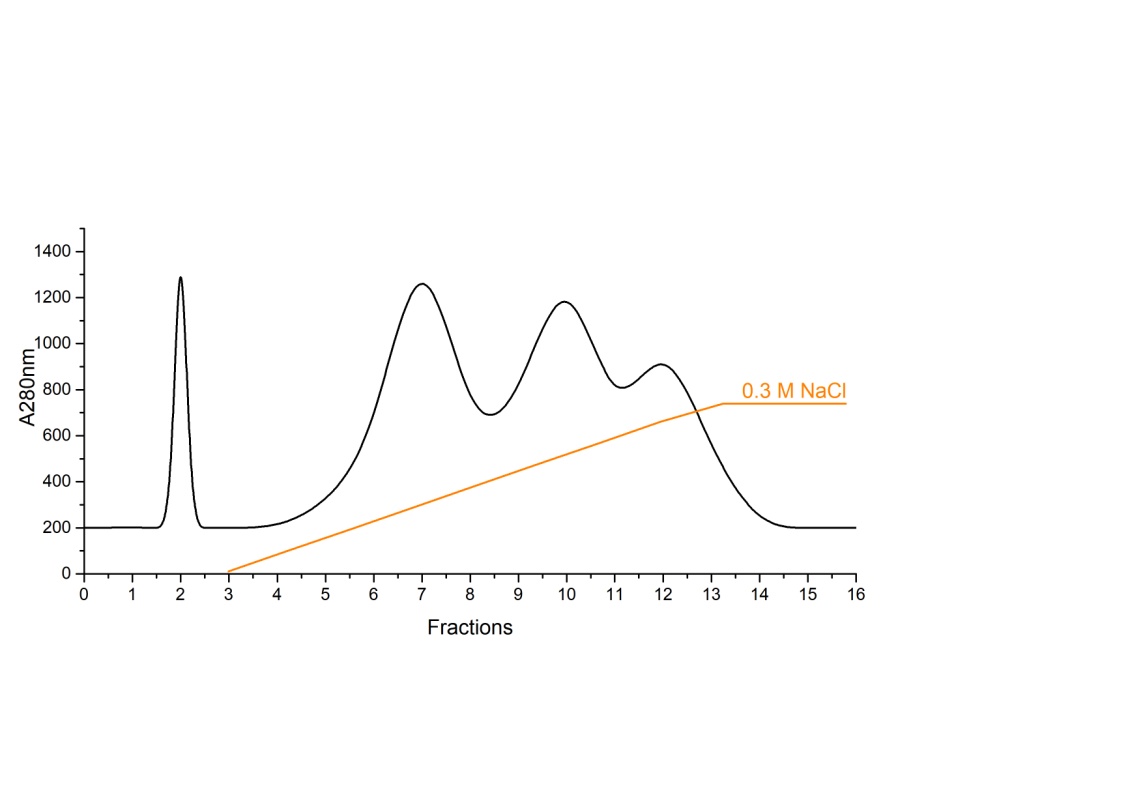


**B**

**
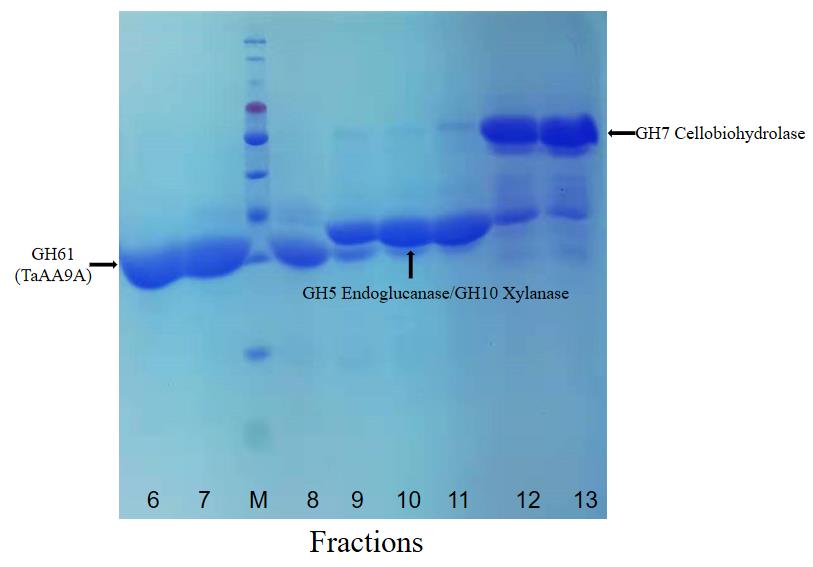
**

**Fig. S3.** **Identification of AA9 LPMOs of thermophilic fungi in the horse gut using LC-MS/MS.** The isolated proteins in the horse gut were digested with trypsin. The extracted-ion chromatograms (EIC) and MaxQuant search results of the isolated protein peptides were shown as follows. Fragmentation *m/z* values agree with the molecular weight of the corresponding fragmentations of the different peptides of the isolated proteins digested with trypsin.

**
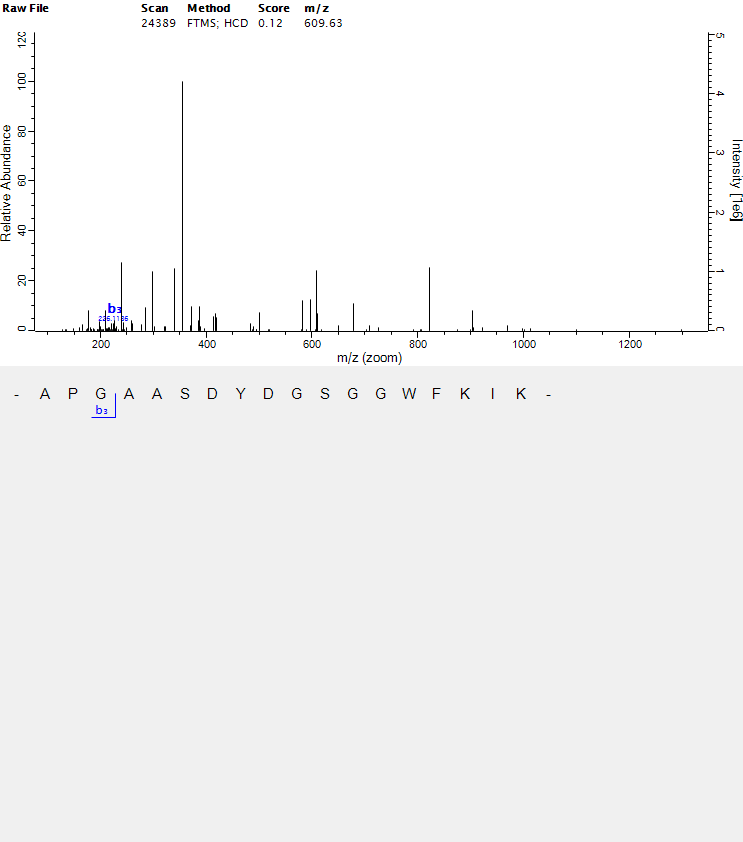
**

**
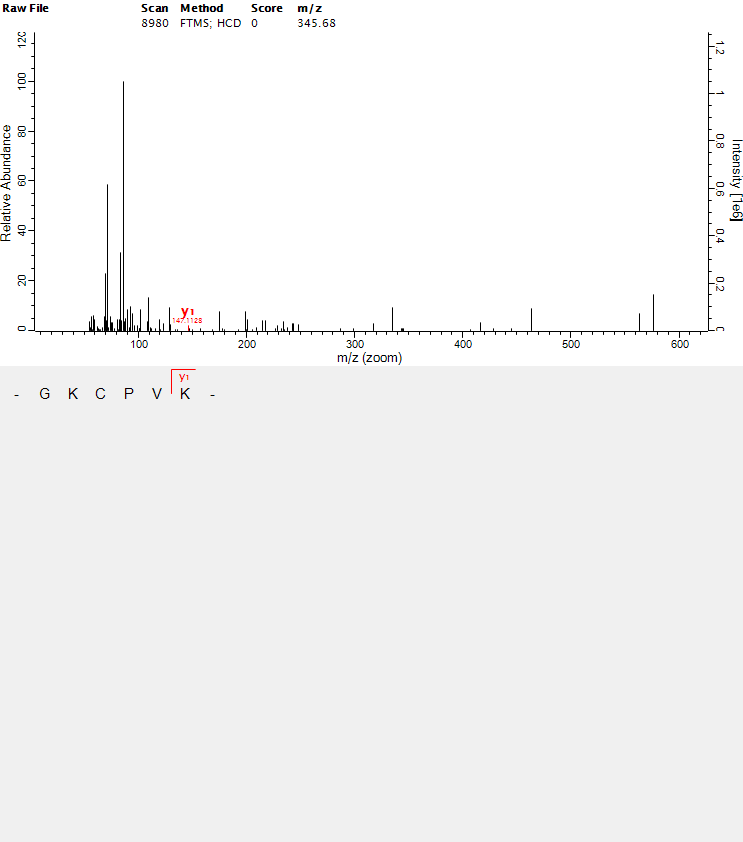
**

**
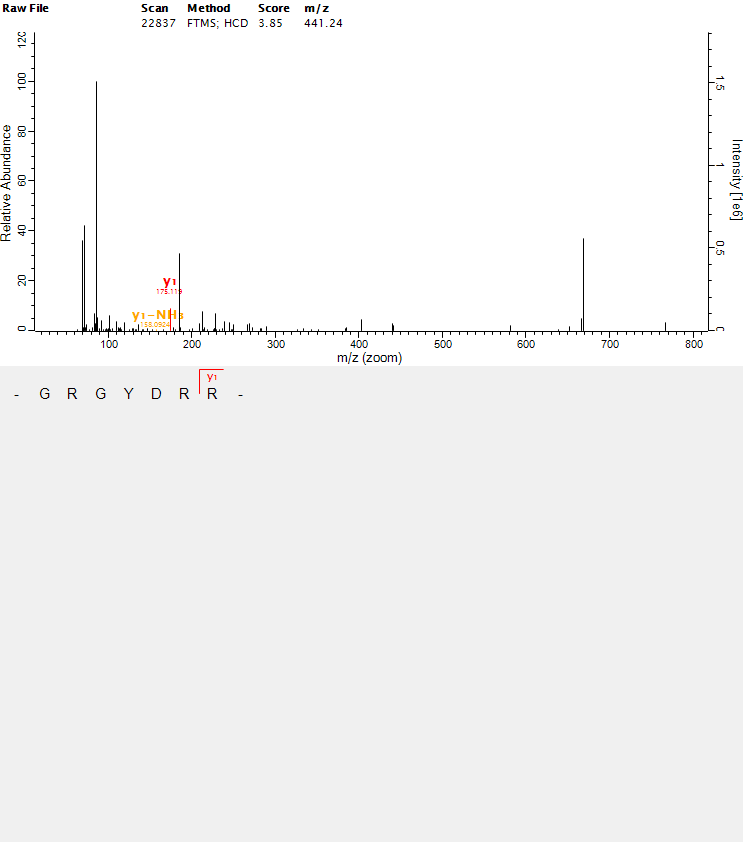
**

**
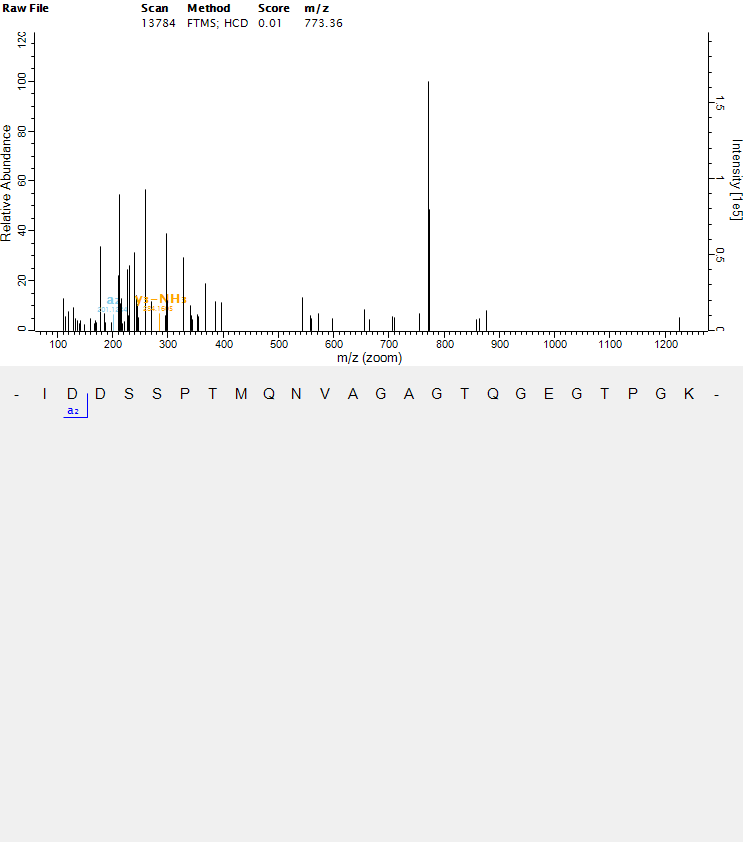
**

**
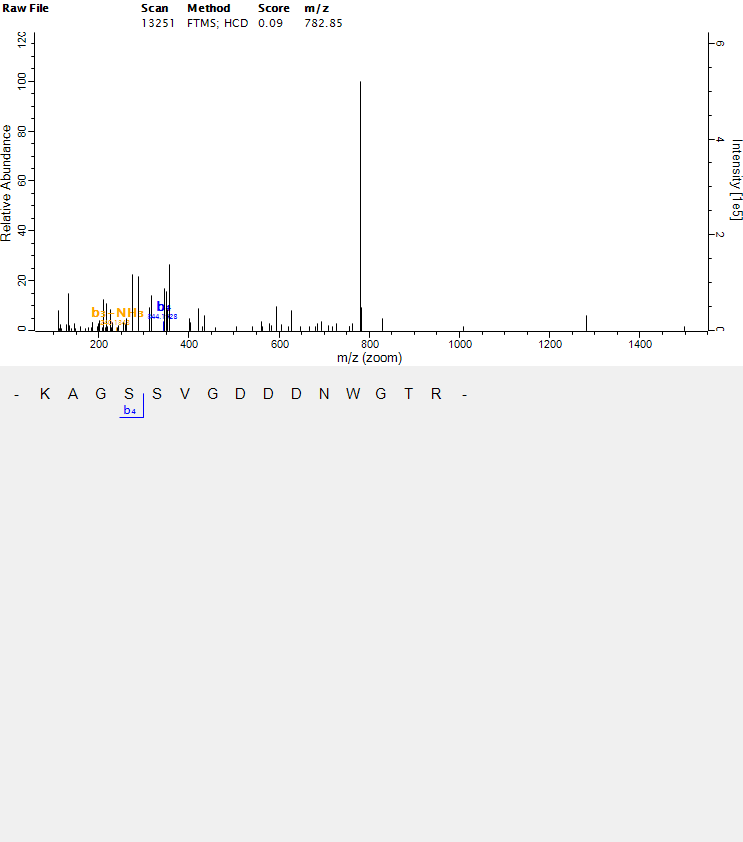
**

**
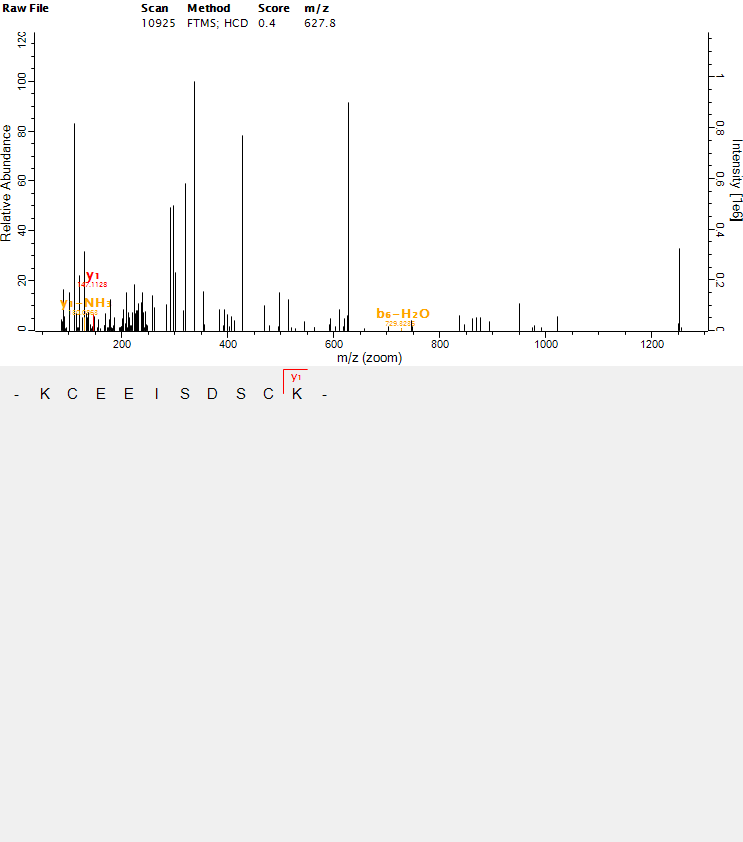
**

**
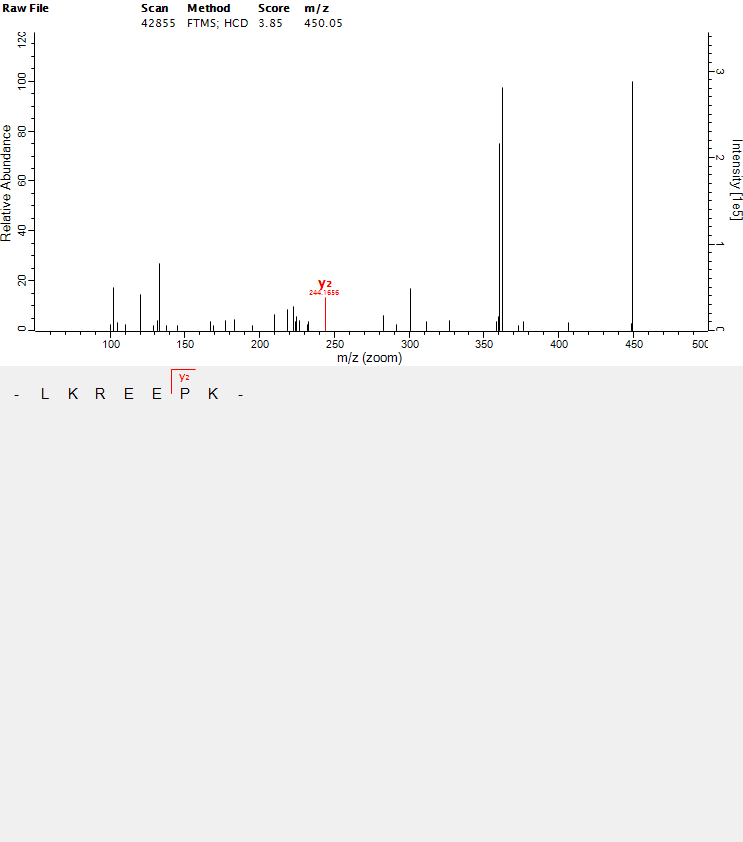
**

**Table S1. ITS sequences of *Chaetomium thermophilum*, *Thermoascus aurantiacus*, *Scytalidium thermophilum*.** The ITS sequences were deposited in a public database of biological sequences like GenBank.

| Scientific name | ITS sequence | Accession number |
| --- | --- | --- |
| *Scytalidium thermophilum* | TTCCTTAGGGGTGACCTGCGGAGGGATCATTACAGAGTTACACAACTCACCAAACCATTGTGAACCGACCCTTCTGTTGCTTCGGCGGGAGGCGCCTCGGCGCCCCTCGGCCGATCCCCCGCCCCCGCGGCGTGGGGGGAGGCTCCCGCCGGAGGAATACCCCAAACCCTTTGCAGCACATGGCCTCTCTGAGTTTATGTACTGAATAAGTCAAAACTTTCAACAACGGATCTCTTGGTTCTGGCATCGATGAAGAACGCAGCGAAATGCGATAAGTAATGTGAATTGCAGAATTCAGTGAATCATCGAATCTTTGAACGCACATTGCGCCCGCCGGTATTCCGGCGGGCATGCCTGTTCGAGCGTCATTTCAACCATCAAGCCCTGGGCTTGTGTTGGGGACCTGCGGCTGCCGCAGGCCCTGAAATGCAGTGGCGGGCTCGCAAGACACACCGAGCGTAGTAGCTTACAACCTCGCTTTGGGAGTGCTGCGGGTGCTGGCCGTAAAGCGACCTTTGCGTCTTTAGCACTAGGTTGACCTCGGATCAGGTAGGAGGACCCGCTGAACTTAAGCATATCAAAAGCCCGGAGGAA | OL762475 |
| *Chaetomium thermophilum* | ATCCTGTAGGGTGACCTGCGGAGGGATCATTAATGAGTTACCACAACTCCCAAACCCATTGTGAACCTACCCTTCACGTTGCTTCGGCGGGTCGGGCGCTGTGCCGCCCCTCCGGGCGACCCGGGGCTCCCCAAAGGCCCCGGGCCCGCCGCCCGCCGGAGGTACGCAAACTCTTGTTCATTGTACGGCCTCTCTGAGTGAAGTACTGAATAAGTCAAAACTTTCAACAACGGATCTCTTGGTTCTGGCATCGATGAAGAACGCAGCGAAATGCGATAAGTAATGTGAATTGCAGAATTCCGTGAATCATCGAATCTTTGAACGCACATTGCGCCCGCCGGTATTCCGGCGGGCATGCCTGTTCGAGCGTCATTTCAACCATCAAGCCCTGGGCTTGTGTTGGGGACCCGCGGCTGCTCGCGGGCCCTGAAAAGCAGTGGCGGGCTCGCTAGTCACACCGAGCGTAGTAGAACTTCTATTCGATCTCGCTCAGGGCGTGCGGCGGGTGCCAGCCGTAAAACCCCAGCTTCTTCTAAGGTTGACCTCGGATCAGGTAGGAGGACCCGCTGAACTTAAGCATATCAAAAGGCAGGAGGAA | OL762476 |
| *Thermoascus aurantiacus* | CTTCCGTAGGGTGACCTGCGGAAGGATCATTACCGAGTGCGGGTCCTCCGGGGCCCAACCTCCCACCCGTGTGTACCGTACCCTGTTGCTTCGGCGGGCCCGCCGCAAGGCCGCCGGGGGGCGTGTCCCGCCCCCGGGCCCGCGCCCGCCGGAGACCCTTCGAACGCTGAGCTTTTGAAGGCGTGCCGTCTGAGTCGCGTGAGAAATCGTGAAAACTTTCAACAACGGATCTCTTGGTTCCGGCATCGATGAAGAACGCAGCGAAATGCGATAAGTAATGTGAATTGCAGAATTCCGTGAATCATCGAATCTTTGAACGCACATTGCGCCCCCTGGCATTCCGGGGGGCATGCCTGTCCGAGCGTCATTGCTGCCCTCAAGCCCGGCTTGTGTGTTGGGCCGCCGTCCCCGCCCGCCGCGGGGGGACGGGCCCGAAAGGCAGCGGCGGCGCCGCGTCCGGTCCTCGAGCGTATGGGGCTTCGTCACCCGCTCTTGCAGGCCCGGCCGGAGCCTCAGCCCGACCCCGCGTCAACATCTTCCAGGTTGACCTCGGATCAGGTAGGGATACCCGCTGAACTTAAGCATATCAATAAGGCGGAGGAA | OL762477 |

**Table S2. Molecular identification of thermophilic fungi from fresh horse feces using subunit 5.8S rDNA gene (ITS1 and ITS4).**

| No | Blast research results | Identity (%) | Length (bp) | Homologue sequcences |
| --- | --- | --- | --- | --- |
| 1 | *C*. *thermophilum* | 100.00% | 569bp | KC342039.1 |
| 2 | *T*. *aurantiacus* | 99.63% | 575bp | JX094778.1 |
| 3 | *S*. *thermophilum* | 99.63% | 561bp | KX912083.1 |

**Tabe S3. List of primers used for PCR in this study*.***

| Primers | Sequence | Purpose |
| --- | --- | --- |
| HiPMO1-F | 5’-CACGGCCATGTCAGCCACATCATT-3’ | ORF cDNA of HiPMO1 |
| HiPMO1-R | 5’-GATGCACTGCGAGTACCAGTCGTT-3’ | ORF cDNA of HiPMO1 |
| CtPMO1-F | 5’-CATGCCATCTTTCAGAAAGTCTCC-3’ | ORF cDNA of CtPMO1 |
| CtPMO1-R | 5’-GCACGTGATGGGAGCCGGCCCAG-3’ | ORF cDNA of CtPMO1 |
| TaAA9A-F | 5’-CATGGCTTCGTTCAGAACATCGTG-3’ | ORF cDNA of TaAA9A |
| TaAA9A-R | 5’-ACCAGTATACAGAGGAGGACCAGG-3’ | ORF cDNA of TaAA9A |
| ITS1 | 5’-TCCGTAGGTGAACCTGCGG-3’ | ITS region |
| ITS4 | 5’-TCCTCCGCTTATTGATATGC-3’ | ITS region |

**Table S4. Data bank of protein sequences of LPMOs from thermophilic fungi.** These sequences are from the publicly available genome data banks (www.fungalgenomics.ca, www.CAZy.org, http://ct.bork.embl.de/).

| Scientific name | Protein ID | Protein Sequence |
| --- | --- | --- |
| *Scytalidium thermophilum* | Scyth2p4_002689 | MKLLPTLAAIAATLATADAHYIFNILYVNGQRMGGEYTYVRRNSNSYFPVFPDILNSNDMRCNVGARPGNTQTATVRAGDRIGFKVFNNEVIEHPGPGFIYMSKAPGSVNNYDGSGDWFKVYETGLCRGGGNVDTNWCSYYKDRLEFTIPPKTPPGEYLVRIEHIGLHEGHVNRAQFYITCAQLKIEGPGGGNPNPLVKIPGIYRANDPGIAYNKWTNNPAPYIMPGPKVWDGN |
| *Scytalidium thermophilum* | Scyth2p4_003909 | MPPPLLATVLSLLAFTRGALSHSHLAHVIINGQLYHGFDPRPNQNNHPARVGWSTTATDDGFVTPGNYSHPDIICHRGGVSPRAHAPVTAGGKVQVQWNGWPIGHVGPILTYIAPCGGLPGAEEGCTGVDKTDLRWTKIDDSMPPFRFTDATKPVSGRAQFPIGQVWATDALVEANNSWSVVIPRNIPPGPYVLRQEIVALHYAAKLNGAQNYPLCLNLWVEKGQQDQGEPFKFDAYDAREFYSEDHPGVLIDVMTMVGPRAVYRIPGPTVASGATRIPHSLQTSAETWVEGTPVAVTRATETVQMEITTTPAGQGAGVRTATPAMPTPTVTKRWKGRFEMGRP |
| *Scytalidium thermophilum* | Scyth2p4_006586 | MPRFTKSIVSALAGASLVAAHGHVTHIVINGVLYPNFDPTSHPYMQNPPTVVGWTAANTDNGFVAPDQFASGDIICHNQATNAGGHAVVAAGDKIWIQWDQWPESHHGPVLDYLASCGSSGCESVNKLDLEFFKIGEKGLIDGSSAPGRWASDELIANNAGWLVQIPADIAPGHYVLRHEIIALHAAGQPNGAQNYPQCFNLLVTGSGTARPQGVKGTALYTPNDKGILAGIYNAPVSYEIPGPALYSGAARNLQQSSSQATSTATALTGDAVPVPTQAPVTTTSSSSADAATTTSTTVQPPQQTTLTTAIATSTAAAAPTTTTGNGNGGNRPFPTRCPGLAGLGFDKRRRQLRAEEGVQVVA |
| *Scytalidium thermophilium* | Scyth2p4_007556 (HiPMO1) | MAPKTSTFLASLTGAALVAAHGHVSHIIVNGVQYRNYDPTTDFYSGNPPTVIGWSALNQDNGFIEPNNFGSPDIICHKSAKPGGGHVTVRAGDKISIVWTPEWPESHVGPVIDYLAACNGNCETVDKTSLRFFKIDGAGYDAAAGRWAADALRANGNSWLVQIPADLKAGNYVLRHEIIALHGAANPNGAQAYPQCINIRVTGGGNNQPSGVPGTQLYKASDPGILFNPWVANPQYPVPGPALIPGAVSSIPQSRSTATATGTATRPGADTDPTGVPPVVTTTSAPAQVTTTSSRTTSLPQITTTFATSTTPPPPAATQSKWGQCGGNGWTGPTLCAPGSSCNKLNDWYSQCI |
| *Scytalidium thermophilum* | Scyth2p4_010865 | MFFRNAATLALAYATTGVSAHALMYGVWVNGVDQGDGRNVYIRTPPNNSPVKDLASPDIVCNVNGGRAVPDFVQASAGDTLTFEWLHNTRGDDIIDRSHLGPIITYIAPFTTGNPTGPVWTKIAEQGFNPSTRRWAVDDLIDNGGKTDFVLPASLAPGKYIIRQEIIAHHESETTFESNPARGAQFYPSCVQIEVSGSGTAVPDQNFDFNTGYTYADPGIHFNIYTSFNSYSIPGPEVWTGASTGGGNGNGNATPTQPTPTPTVTPTPIETAQPVTTTTTSTRPFPTRCPGRRLKREEPKA |
| *Scytalidium thermophilum* | Scyth2p4_007651 | MKLLAPLMLAGAASAHTIFTSLEVDGRNYGTGNGVRVPSYNGPVEDVTSNSIACNGPPNPTSPTDTVITVQAGQNVTAIWRYMLNTQGTSPNDIMDSSHKGPTLAYLKKVNDARTDSGVGDGWFKIQHDGFDGTTWGTERVIFGQGRHTIKIPECIEPGQYLLRAEMIALHGAQNYPGAQFYMECAQLNIVGGTGTKKPSTVSFPGAYKGTDPGVKISIWWPPVTNYVIPGPDVFKC |
| *Thermomyces lanuginosus* | [Thela2p4_000810](http://genome.fungalgenomics.ca/new_gene_model_pages/gene_model_page.php?gmid=Thela2p4_000810) | MKGSSAASVLLTFLAGISRTSAHGYVSNLVINGVYYRGWLPGEDPYNPDPPIGVGWETPNLGNGFVTPSEASTDAVICHKEATPARGHVSVKAGDKIYIQWQPNPWPDSHHGPVLDYLAPCNGPCESVDKTSLRFFKIDGVGLIDGSSPPGYWADDELIANGNGWLVQIPEDIKPGNYVLRHEIIALHSAGNPDGAQLYPQCFNLEITGSGTVEPEGVPATEFYSPDDPGILVNIYEPLSTYEVPGPSLIPQAVQIEQSSSAITATGTPTPA |
| *Thermomyces lanuginosus* | Thela2p4_003424 | MKGSTTASLLLPLLASVTRTSAHGFVSNLVINGVFYRGWLPTEDPYKADPPIGVGWETPNLGNGFVLPEEASTDAIVCHKEAEPARGYASVAAGDKIYIQWQPNPWPESHHGPVIDYLAPCNGDCSTVNKTSLEFFKIDGVGLIDGSSPPGKWADDELIANGNGWLVQIPEDIKPGNYVLRHEIIALHEAFNQNGAQIYPQCFNLQITGSGTVEPEGTPATELYSPTDPGILVDIYNPLSTYVVPGPTLIPQAVEIEQSSSAVTATGTPTPAAA |
| *Thermomyces lanuginosus* | Thela2p4_002033 | MAFSNIFSRPARLVLSATVFLSLAQAHTVMTTLYVDGENQGDGVCIRMNMDGYTSNYFVSPVTSKDIACGVDGEKGVSRVCPAKTSSVLTFEFREDADDVNSRPLDESHKGPAAVYMKKVSSATDSNNAAGDGWFKIWESVYDSENDKWGTTKMIENNGHISVRVPEDIEGGYYLVRTELLALHAATANPPDPQFYVGCAQVFIESNVTNPSRPETVFIGEGTYTLDNPALTFNIYDKPMALPYPPLGPPVYKPKSNPDTTEGNSQEQTEGLKPEGCIFVNGNWCGFEVPSYNNEEECWAASDDCWRQSDNCWAETQPTGYGYCTEWSKKCEEISDSCKNSRFPGPPNAGQDITPEWKKLEEGGTEIFY |
| *Thermomyces lanuginosus* | Thela2p4_000154 | MAFSTVTVFVTFLAFISIASAHGFVTKITVLGDNNKDYPGFDPSTPKEVPPGLDVAWSTSASDQGYMSSSNASYHSKDFICHRNAKPAPDAAQVHAGDKVQLHWTQWPGPEDHQGPILDYLASCNGPCSNVEKASLKWTKIDEAGRFPNGTWATDLLRNGGNTWNVTIPSDLAPGEYVLRNEIIALHSARNMGGAQHYMQCVNLNVTGTGHRELQGVSAAEFYNPTDPGILINVWQTQSLSSYHIPGPTLLAADTGNDGGHSASSTLATVTSRRLSTPSDAMPGNGSYGAISPPLKPAKGFHPVCNARFRHGSTFTLTTLVAPPART |
| *Thermoascus aurantiacus* | ABW56451.1 | msfskiiatagvlasaslvaghgfvqnividgkkyyggylvnqypymsnppeviawsttatdlgfvdgtgyqtpdiichrgakpgaltapvspggtvelqwtpwpdshhgpvinylapcngdcstvdktqleffkiaesglinddnppgiwasdnliaannswtvtipttiapgnyvlrheiialhsaqnqdgaqnypqcinlqvtgggsdnpagtlgtalyhdtdpgiliniyqklssyIipgpplytg |
| *Thermoascus aurantiacus* | ACS05720.1 | msfskiiatagvlasaslvaghgfvqnividgkkyyggylvnqypymsnppeviawsttatdlgfvdgtgyqtpdiichrgakpgaltapvspggtvelqwtpwpdshhgpvinylapcngdcstvdktqleffkiaesglinddnppgiwasdnliaannswtvtipttiapgnyvlrheiialhsaqnqdgaqnypqcinlqvtgggsdnpagtlgtalyhdtdpgiliniyqklssyIipgpplytg |
| *Thermoascus aurantiacus* | CCP37673.1 | chkgaepgalsakvaaggtvelqwtdwpeshkgpvidylaacngncstvdktkleffkidesglidgssapgtwasdnliannnswtvtipstmlp |
| *Thermoascus aurantiacus* | AGO68294.1(TaAA9A) | msfskiiatagvlasaslvaghgfvqnividgkkyviarrnqypymsnppeviawsttatdlgfvdgtgyqtpdiichrgakpgaltapvspggtvelqwtpwpdshhgpvinylapcngdcstvdktqleffkiaesglinddnppgiwasdnliaannswtvtipttiapgnyvlrheiialhsaqnqdgaqnypqcinlqvtgggsdnpagtlgtalyhdtdpgtliniyqklssyiIpgpplytg |
| *Thermothielavioides terrestris* | AEO62422.1 | mkfslvsllayglsveahsifqrvsvngqdqglltglrapsnnnpvqdvnsqnmicgqsgsksqtvinvkagdrigslwqhviggaqfsgdpdnpiahshkgpvmaylakvdnaasasqtglkwfkiwqdgfdtssktwgvdnliknngwvyfhlpqclapgqyllrvevlalhsayqqgqaqfyqscaqinvsgsgsfspsqtvsipgvysatdpsiliniygstgqpdnggkaynppgpapisc |
| *Thermothielavioides terrestris* | AEO67662.1 | mklttsvallaaagaqahytfpqtdingqlsgewvtirettnhyshgpvtdvtsdqircyelnpgtpapqiatvqaggtvtftvdpsiqhpgplqfymakapsgqtaatfqgtgnvwfkiyedgpsglgtsnitwpssgktevsvkipsciapgdyllrvehialhsastvggaqfylacaqltvtggtgtlntgelvafpgaysatdpgilfqlywpiptsytnpgpapvsc |
| *Thermothielavioides terrestris* | AEO64605.1 | mklssqlaaltlaaasvsghyifeqiahggtkfppyeyirrntnynspvtslssndlrcnvggetagnttvldvkagdsftfysdvavyhqgpislymskapgsvvdydgsgdwfkihdwgptfsngqaswplrdnyqyniptcipngeyllriqslaihnpgatpqfyiscaqvrvsgggsaspsptakipgafkatdpgytaniynnfhsytvpgpavfqc |
| *Thermothielavioides terrestris* | AEO69044.1 | mksftiaalaalwaqeaaahatfqdlwidgvdygsqcvrlpasnspvtnvasddircnvgtsrptvkcpvkagstvtiemhqqpgdrscaneaiggdhygpvmvymskvddavtadgssgwfkvfqdswaknpsgstgdddywgtkdlnsccgkmnvkipediepgdyllraevialhvaassggaqfymscyqltvtgsgsatpstvnfpgaysasdpgilinihapmstyvvpgptvyaggstksagsscsgceatctvgsgpsatltqptstatatsapggggsgctaakyqqcggtgytgcttcasgstcsavsppyysqcl |
| *Thermothielavioides terrestris* | AEO64593.1 | mlltsvlgsaallasgaaahgavtsyiiagknypgyqgfspanspnviqwqwhdynpvlscsdsklrcnggtsatlnataapgdtitaiwaqwthsqgpilvwmykcpgsfsscdgsgagwfkideagfhgdgvkvfldtenpsgwdiaklvggnkqwsskvpeglapgnylvrhelialhqannpqfypecaqvvitgsgtaqpdasykaaipgycnqndpnikvpindhsipqtykipgppvfkgtaskkardfta |
| *Thermothielavioides terrestris* | AEO65580.1 | mrfdalsalalaplvaghgavtsyiiggktypgyegfspasspptiqyqwpdynptlsvtdpkmrcnggtsaelsapvqagenvtavwkqwthqqgpvmvwmfkcpgdfsschgdgkgwfkidqlglwgnnlnsnnwgtaivyktlqwsnpipknlapgnylirhellalhqantpqfyaecaqlvvsgsgsalppsdylysipvyapqndpgitvdiynggltsytppggpvwsgfef |
| *Thermothielavioides terrestris* | AEO66274.1 | mktftallaaaglvaghgyvdnatiggqfyqfyqpyvdpylatlpdrvsrsipgngpvtdvtlidlqcnanstpaklhataaagsdvilrwtlwpeshvgpvitymarcpdtgcqdwmpgtsavwfkikeggrdgtsntwadtplmtaptsytytipsclkkgyylvrheiialhaaytypgaqfypgchqlnvtgggstvpssglvafpgaykgsdpgitydaykaqtyqipgpavftc |
| *Thermothielavioides terrestris* | AEO67396.1 | mmpslvrfsmglatafaslstahtvfttlfingvdqgdgtcirmakkgsvcthpiaggldspdmacgrdgqqavaftcpapagsklsfefrmwadasqpgsidpshlgstaiylkqvsnissdsaagpgwfkiyaegydtaakkwateklidnggllsielpptlpagyylarseivtiqnvtndhvdpqfyvgcaqlfvqgppttptvppdrlvsipghvhasdpgltfniwrddpsktaytvvgpapfsptaaptptstntngqqqqqqqqaikqtdgvipadcqlknanwcgaevpayadeagcwassadcfaqldacytsapptgsrgcrlwedwctgiqqgcragrwrgpppfhgEgaaaev |
| *Thermothielavioides terrestris* | AEO68157.1 | mklsvaiavlasalaeahytfpsigntadwqyvrittnyqsngpvtdvtsdqircyernpgtgaqgiynvtagqtinynakasishpgpmsfyiakvpagqtaatwdgkgavwtkiyqdmpkfgssltwptmgaksvpvtiprclqngdyllraehialhsassvggaqfylscaqltvsggsgtwnpknrvsfpgaykatdpgiliniyypvptsysppgppaetc |
| *Thermothielavioides terrestris* | AEO68577.1 | mallllaglailagpahahgglanytvgntwyrgydpftpaadqigqpwmiqrawdsidpifsvndkalacntpataptsyipiragenitavywywlhpvgpmtawlarcdgdcrdadvnearwfkiweagllsgpnlaegmwyqkafqnwdgspdlwpvtipaglksglymirheilsihvedkpqfypecahlnvtgggdllppdeflvkfpgaykednpsikiniysdqyanttnytipggpiwdg |
| *Thermothielavioides terrestris* | AEO68763.1 | mppalpqllttvltaltlgstalahshlayiivngklyqgfdprphqanypsrvgwstgavddgfvtpanystpdiichiagtspaghapvrpgdrihvqwngwpvghigpvlsylarcesdtgctgqnktalrwtkiddssptmqnvagagtqgegtpgkrwatdvliaannswqvavpaglptgayvlrneiialhyaarkngaqnyplcmnlwvdasgdnssvaattaavtagglqmdaydargfykendpgvlvnvtaalssyvvpgptvaagatpvpyaqqspsvstaagtpvvvtrtsetapytgamtptvaarmkgrgydrrg |
| *Thermothielavioides terrestris* | AEO71031.1 | mqllvglllaavaarahytfprlvvngqpedkdwsvtrmtknaqskqgvqdptspdircytsqtapnvatvpagatvhyistqqinhpgptqyylakvpagssaktwdgsgavwfkisttmpyldnnkqlvwpnqntyttvnttipadtpsgeyllrveqialhlasqpngaqfylacsqiqitgggngtpgplvalpgayksndpgilvniysmqpgdykppgppvwsg |
| *Thermothielavioides terrestris* | AEO67395.1 | mrttfaaalaafaaqevaghaifqqlwhgsscvrmplsnspvtnvgsrdmicnagtrpvsgkcpvkaggtvtvemhqqpgdrscnneaiggahwgpvqvylskvedastadgstgwfkifadtwskkagssvgdddnwgtrdlnaccgkmqvkipadipsgdyllraealalhtagqvggaqfymscyqitvsgggsaspatvkfpgaysandpgihinihaavsnyvapgpavysggttkvagsgcqgcentckvgssptatapsgksgagsdggagtdggssssspdtgsacsvqaygqcggngysgctqcapgytckavsppyysqcapss |
| *Thermothielavioides terrestris* | AEO69043.1 | mklylaaflgavatpgafahqihgillvngtetpewkyvrdvawegayepekypnteffktppqtdinnpnitcgrnafdsasktetadilagsevgfrvswdgngkygvfwhpgpgqiylsrapnddledyrgdgdwfkiatgaavsntewllwnkhdfnftipkttppgkylmrieqfmpstveysqwyvncahvniigpgggtptgfarfpgtytvddpgikvplnqivnsgelpqdqlrlleykppgpalwtg |
| *Thermothielavioides terrestris* | AEO65532.1 | mkglfsaaalslavgqasahyifqqlsingnqfpvyqyirkntnynspvtdltsddlrcnvgaqgagtdtvtvkagdqftftldtpvyhqgpisiymskapgaasdydgsggwfkikdwgptfnadgtatwdmagsytyniptcipdgdyllriqslaihnpwpagipqfyiscaqitvtgggngnpgptalipgafkdtdpgytvniytnfhnytvpgpevfscngggsnppppvssstpatttlvtstrttsstssastpastggctvakwgqcggngytgcttcaagstcskqndyysqcl |
| *Thermothielavioides terrestris* | AEO68023.1 | mkglsllaaasaatahtifvqlesggttypvsygirdpsydgpitdvtsdslacngppnpttpspyiinvtagttvaaiwrhtltsgpddvmdashkgptlaylkkvddaltdtgigggwfkiqeagydngnwatstvitnggfqyidipacipngqyllraemialhaastqggaqlymecaqinvvggsgsaspqtysipgiyqatdpglliniysmtpssqytipgpplftcsgsgnngggsnpsggqtttakpttttaatttssaaptssqggssgctvpqwqqcggisftgcttcaagytckylndyysqcq |
| *Thermothielavioides terrestris* | ACE10234.1 | mlangaivflaaalgvsghytwprvndgadwqqvrkadnwqdngyvgdvtspqircfqatpspapsvlnttagstvtywanpdvyhpgpvqfymarvpdgedinswngdgavwfkvyedhptfgaqltwpstgkssfavpippciksgyyllraeqiglhvaqsvggaqfyiscaqlsvtgggsteppnkvafpgaysatdpgiliniyypvptsyqnpgpavfscmlangaivflaaalgvsghytwprvndgadwqqvrkadnwqdngyvgdvtspqircfqatpspapsvlnttagstvtywanpdvyhpgpvqfymarvpdgedinswngdgavwfkvyedhptfgaqltwpstgkssfavpippciksgyyllraeqiglhvaqsvggaqfyiscaqlsvtgggsteppnkvafpgaysatdpgiliniyypvptsyqnpgpavf sc |
| *Thermothielavioides terrestris* | AEO71030.1 | mlangaivflaaalgvsghytwprvndgadwqqvrkadnwqdngyvgdvtspqircfqatpspapsvlnttagstvtywanpdvyhpgpvqfymarvpdgedinswngdgavwfkvyedhptfgaqltwpstgkssfavpippciksgyyllraeqiglhvaqsvggaqfyiscaqlsvtgggsteppnkvafpgaysatdpgiliniyypvptsyqnpgpavfsc |
| *Thermothielavioides terrestris* | AEO64177.1 | mpsfasktllstlagaasvaahghvsnivingvsyqgydptsfpymqnppivvgwtaadtdngfvapdafasgdiichknatnakghavvaagdkifiqwntwpeshhgpvidylascgsascetvdktkleffkidevglvdgssapgvwgsdqliannnswlveipptiapgnyvlrheiialhsaenadgaqnypqcfnlqitgtgtatpsgvpgtslytptdpgilvniysapitytvpgpalisgavsiaqsssaitasgtaltgsatapaaaaatttsttnaaaaatsaaaaagtsttttsaaavvqtssssssapssaaaaatttaaasarptgcssgrsrkq prrhardmvvargaeean |
| *Thermothielavioides terrestris* | CAG27576.1 | mklttsvallaaagaqahytfpqtdingqlsgewvtirettnhyshgpvtdvtsdqircyelnpgtpapqiatvqaggtvtftvdpsiqhpgplqfymakapsgqtaatfqgtgnvwfkiyedgpsglgtsnitwpssgktevsvkipsciapgdyllrvehialhsastvggaqfylacaqltvtggtgtlntgelvafpgaysatdpgilfqlywpiptsytnpgpapvsc |
| *Thermothielavioides terrestris* | ACE10232.1 | mrfdalsalalaplvaghgavtsyiiggktypgyegfspasspptiqyqwpdynptlsvtdpkmrcnggtsaelsapvqagenvtavwkqwthqqgpvmvwmfkcpgdfssshgdgkgwfkidqlglwgnnlnsnnwgtaivyktlqwsnpipknlapgnylirhellalhqantpqfyaecaqlvvsgsgsalppsdylysipvyapqndpgitvdiynggltsytppggpvwsgfefmrfdalsalalaplvaghgavtsyiiggktypgyegfspasspptiqyqwpdynptlsvtdpkmrcnggtsaelsapvqagenvtavwkqwthqqgpvmvwmfkcpgdfssshgdgkgwfkidqlglwgnnlnsnnwgtaivyktlqwsnpipknlapgnylirhellalhqantpqfyaecaqlvvsgsgsalppsdylysipvyapqndpgitvdiynggltsytppggpvwsgfef |
| *Thermothielavioides terrestris* | ACE10233.1 | mlltsvlgsaallasgaaahgavtsyiiagknypgyqgfspanspnviqwqwhdynpvlscsdsklrcnggtsatlnataapgdtitaiwaqwthsqgpilvwmykcpgsfsscdgsgagwfkideagfhgdgvkvfldtenpsgwdiaklvggnkqwsskvpeglapgnylvrhelialhqannpqfypecaqvvitgsgtaqpdasykaaipgycnqndpnikvpindhsipqtykipgppvfkgtaskkardftamlltsvlgsaallasgaaahgavtsyiiagknypgyqgfspanspnviqwqwhdynpvlscsdsklrcnggtsatlnataapgdtitaiwaqwthsqgpilvwmykcpgsfsscdgsgagwfkideagfhgdgvkvfldtenpsgwdiaklvggnkqwsskvpeglapgnylvrhelialhqannpqfypecaqvvitgsgtaqpdasykaaipgycnqndpnikvpindhsipqtykipgppvfkgtaskkardfta |
| *Thermothielavioides terrestris* | ACE10235.1 | mkglfsaaalslavgqasahyifqqlsingnqfpvyqyirkntnynspvtdltsddlrcnvgaqgagtdtvtvkagdqftftldtpvyhqgpisiymskapgaasdydgsggwfkikdwgptfnadgtatwdmagsytyniptcipdgdyllriqslaihnpwpagipqfyiscaqitvtgggngnpgptalipgafkdtdpgytvniytnfhnytvpgpevfscngggsnppppvssstpatttlvtstrttsstssastpastggctvakwgqcggngytgcttcaagstcskqndyysqclmkglfsaaalslavgqasahyifqqlsingnqfpvyqyirkntnynspvtdltsddlrcnvgaqgagtdtvtvkagdqftftldtpvyhqgpisiymskapgaasdydgsggwfkikdwgptfnadgtatwdmagsytyniptcipdgdyllriqslaihnpwpagipqfyiscaqitvtgggngnpgptalipgafkdtdpgytvniytnfhnytvpgpevfscngggsnppppvssstpatttlvtstrttsstssastpastggctvakwgqcggngytgcttcaagstcskqndyysqcl |
| *Thermothielavioides terrestris* | ACE10231.1 | mksftiaalaalwaqeaaahatfqdlwidgvdygsqcvrlpasnspvtnvasddircnvgtsrptvkcpvkagstvtiemhqqpgdrscaneaiggdhygpvmvymskvddavtadgssgwfkvfqdswaknpsgstgdddywgtkdlnsccgkmnvkipediepgdyllraevialhvaassggaqfymscyqltvtgsgsatpstvnfpgaysasdpgilinihapmstyvvpgptvyaggstksagsscsgceatctvgsgpsatltqptstatatsapggggsgctaakyqqcggtgytgcttcasgstcsavsppyysqcl |
| *Chaetomium thermophilum* | EGS19579.1 | MPSFVSKTLISALAGAASVAAHGHVKNFVINGLSYQAYDPTVFPYMQNPPIVAGWTSANTDNGFVGPEDYSNPDIICHKSATNAKGHAVIKAGDSVYIQWDTWPESHHGPVIDYLASCGSAGCETVDKAQLEFFKIAEAGLIDGSQAPGKWAADQLIAQNNSWLVTIPENIKPGFYVLRHEIIALHSAGQTNGAQNYPVCINLEVTGGGSDLPSGVKGTELYKPTDPGILINIYQPLSSYTIPGPALMPGAKPVTQRTSAIIGSTTAITGTATAAPAAPTSTAAATTTTSANANPIPTITLRTSTIAPQPTAAPTQTPTSRVGQPPRPTRCPGLDNLKRARRHARDLAAH |
| *Chaetomium thermophilum* | EGS20667.1 | MVKAKKSAFLATVAGASLVAAHGYVNGIVVNGVYYRNYNPSVDWYRGNNQETLIGWRAENTDNGFVEPNKFNTADIICHRQAVNAKGYATVKAGDKINIKWDPIWPESHVGPVIDYLADCNGDCSTVSKNSLRFFKIDGAGYDKAKGKWAADVLRENGNSWMVQIPADLKPGHYVLRHEIIALHGAANPNGAQAYPQCINIKVEGSGSNSPSGVAGTSLYTANDPGILFNPWVSNIDYPVPGPALIPGAVSSIQQSTSAATRTASATPYAGGAVPTTTQGGSQSTPTALPTTTLVTTTAVPITTAPPAGPTQSKWGQCGGSGYTGPTLCAPGSNCQVINPWYHQCV |
| *Chaetomium thermophilum* | EGS20367.1 | MPPSTSSLLSILLTLSFTLIPGPVFAHSHLSHIVVNGALYHGYDPRSPVKNPDSNKTYNNPRPNHPGSVAWSYAAFDDGFVAPENYSHPDIICHIGATSPKAHAPVRPGDLIHIQWNGWPVGHVGPILTYIAPCKSQSGCAGVDKTTLRWTKIDESRPVLEILPNNENRWDVEHGRAGIVGKRWATDVMVAANNSWQVEVPRGLARGAYVMRHEIIALHFAAKRGGAQNYPVCFNLWIEGEKEGSVTLDGYDAREFYRDDHIGVWVNVTAPALTSYIIPGPTIASWAMPVPYAQQTSMLLRSEGTPVVVTRSTETVLWTAEVTPTPTGQAVRHRYHLRG |
| *Chaetomium thermophilum* | EGS18626.1(CtPMO1) | MKLSLASLLTAALSVQGHAIFQRVSINGQDHGQLTGLRAPNTNYPVEDVNSQDIICGQSGHRSSTILNVRAGDRIGTFWGHVIGGAQFPGDPDNPIARSHKGPIQVYLAKVDNAASASHTGLRWFKIWSEGFDVGSKRWGVDTMINNGGWYYFNLPQCIADGQYLMRVELLALHSAGNYRGAQFYQSCAQINVSGGGSFTPSQTVSFPGAYSQNDPGILVNIYGLTGQPDNGGKPYQAPGPAPITC |
| *Chaetomium thermophilum* | EGS21628.1 | MILQPAFGIVALLAAEAAAHGAVTSYIIAGKNYPGYQGYSPANSPNVIQWQWPSYDPIMSCSDPKLRCNGGRGATLWAEANPGDEVTAIWQQWTHSQGPIIVWMYKCPGEFSTCDGSGPGWFKIDEAGFNGDGKTVFLDTERPSGWEIAKLVGGNKGWTSKIPQGLAPGNYLIRHELIALHQANAPQFYPECAQIKVLGSGTAEPPASYKVSIPGYCKDSDPNIRVPINDHSIPQTYTIPGPPVWNGVGSTKKARDFTA |
| *Chaetomium thermophilum* | EGS21609.1 | MRLQSLALAATSLTALLPDVASAHYIFQQFSANGVTYPPWKYIRRNTNPAWLQNGPVTDLSSTDLRCNVGGQVSNGTETITLAAGSEFTFTLDTAVYHAGPISLYMSKAPNKVEDYDGSGPWFKIHDWGPSGNSWPLRISYTYNIPKCIPNGEYLLRIQSLGLHNPGAAPQFYISCAQVKVINGGNANPSPTAYIPGAFKATDPGYTVNIYNTNLANYVVPGPRPFTC |
| *Chaetomium thermophilum* | EGS17857.1 | MLSLPLTTLTLTLLPVVSAHTMLYGVSVNSEPLLDGRNRSIRTPLTNSPVKDLASPDLVCNTRGGVPVPEFVEVNAGDTLTFRWFHWNPDDPNDILDPSHKGAILTYIAEYTEGDGRGPRWTKIHQEGFDGGEWATIKMRANGGRVQVELPRNLAPGRYLIRQELLALHMADFRGDDPEHPDKGAESYPNCVQVEVGGNGKARPDQGFDFNEGYTYGDKGLFFNIYIPFEKYTPPGPVVWTGE |
| *Chaetomium thermophilum* | EGS21363.1 | MQYFQMLLGAGTATIAASDRLSFVAVSAITHFGPVSYMARVPNNADINIWEPTGNVWFKVSKINVFPSPNGGSLTSGEAIWLAYNTDKKSVELTILKSIPSGKYIVRVDSIALHQT |
| *Chaetomium thermophilum* | EGS17840.1 | MFPLTHAGFAPGSGNLYGFKSQNGLKAVKLFLSRTIVNGKQTGRGYTYVRRNTNSYNPAFQDLSKEDLRCNVGSKPGDNVKTLEVNTGDRVGFKVFNNELIEHTGPSFVYMSKVPYGVAGA |
| *Chaetomium thermophilum* | EGS21285.1 | MRFPVAFTVGLATAFASLASAHTCFTTLFINDVNQGDGTCVRMPKDGSLSTHPIYGYDNPDLACGRDGNIPVAFTCPAPAGAKLTFEFRMWADLSQPGAIDAGHKGPIAVYLKRVSNITIDSAVGPGWFKIYEEGFDTATNMWAVTKLNASQGLLSINLPSGLPTSYYLIRTEMIALHNVTSNAVSPQPYVGCAQLFIQSSVPPETAIPSEKTVSIPGHLSPSDPGLNFNVYRGEGKESSYRVPGPAVYFPTAPPGNNKAQNPTIQQTDGLIPDNCLVKNANWCGVEVPRYTNQAGCWASAENCWNQLEVCYKTAPPSGNKGCRVWEEQKCKVLQQACSSGQWQGPPNEGQKLTAVVDSPIPGGKLPDPVNAGQQGEVVSGGGGSSSGSGNTGGGQATTFIVSTTSAVPTSVVTSSPTPIPNDEVAPTPTVSPPPRGGKGKGYRPHCKSTRKQQQRRWVVYEPMA |
| *Chaetomium thermophilum* | EGS19451.1 | MKFTTPLALLAVVGVQAHYTFPRTKVNGVLSGEYETVRLTANHWSHGPVTDVTSQEMTCFEKNPGTPAPKTITVQAGNNVTFTVDSNIGHPGPLHFYMAKVPAGQTAATFNGKGPVWFKIYQDGPGGLGTSSLTWPSYGKTEVSVQIPHCIQDGDYLLRVEHIALHSASSIGGAQLYIACAQLTVTGGTGTLNTGQLVSFPGAYKATDPGILFQLYWPPPTSYINPGPAPVKC |
| *Chaetomium thermophilum* | EGS23404.1 | MKPFSLVALATSASAHAIFQRVSVNGVDQGQLVGIRAPSSNFPIENVNHPDFACNTNIVFKDNNVIKIPAGARVGAWWGHEIGGAAGPNDPDHPIAKSHKGPIQVYLAKVDNAATASDKNLQWFKIAERGLNNGVWAVDEMIANNGWHYFDMPQCIAPGHYLMRVELLALHNAFAPGGSQFYMECAQIEVTGSGTHTGSDFVSFPGAYSATHPGITINIYDNFGQPTNGGRPYEIPGPRPISCANAPSNPQPQQPTTTAQPSQPTPTNGGGSGSVPLWGQCGGRGYTGPTTCAQGTCKVQNEWYSQCIP |
| *Chaetomium thermophilum* | EGS17558.1 | MKLLAPLALVGAASAHTIFVSLEVNGVNHGVGNGVRVPSYNGPIEDVTSNSIACNGPPNPTTPTDKVITVQAGSEVTAIWRYMLNSQGSAPNDVMDSSHKGPTMAYLKKVNNATTDSGVGDGWFKIQEDGFDGTTWGTERVINGQGRHKIKIPECIEPGQYLLRAEMLALHGASNYPGAQFYMECAQLNIVGGTGTKKPSTVSFPGAYSGNDPGVKINIWWPPVTSYKVPGPPVFTC |
| *Chaetomium thermophilum* | EGS23697.1 | MKKLLSAAALSLAISDVSAHYIFQQLAINGNKYPVYKYIRKNTNLNHPVIDLDSNDLRCNVGASGADTETITVRAGDTFTFYTDTPVYHQGPISVYMSKAPGKASDYDGSGGWFKIKDWGPTFNGGSATWPLTDNYSGTIPSCIPDGDYLLRIQSLGIHNPWPAGIPQFYISCAQITVTGGGNGTPGPQVLIPGAFKETDPGYTVNIYTNFKNYTVPGPEIFSCNYNGPVPSSPSNPSNPSNPTTSSNSPNLPVTTTLITSTTKSPVTTTTSSTRPVCTPVAKWGQCGGIGYTGCTECAGSTCTKINDWYHQCI |
| *Chaetomium thermophilum* | EGS20384.1 | MKTLSLAALAALWAQKAAAHAMFQQLWVDGVDYGTQCARVPGSNSPVTNVNSPEIRCNAYPSPAKGKCPVKAGSIVTIEMHAQPGDRDCSKEAMGGAHHGPVLVYMTKVSDAATADGSTGWFKIFEDGWAKNPSGKVGDDDFWGTKDLNTCCGKMDVKIPEDIPSGDYLLRAEAIALHVGGQPGGAQLYMTCYQLTVEGGGNANPATVKFPGAYSATDPGILVNIHSAMNNYVVPGPPVYSGGTTKRAGSGCTGCEQTCKVGSSPSGTVSAIPVGTGAPNGGGGCYAQKYQQCGGQGYTG  CTQCVPGSTCQAISPPYYSQCV |
| *Chaetomium thermophilum* | EGS24042.1 | MLATAFLLLGAALNGVTAHYTFPRVQNGFDWQYVRRADNFQSNGFVADVNSQQIRCFQNIHSPAQATLNVTAGSTVTYYSNQAIFHPGPMSFYMARVPDGQDINSWKGEGAVWFKIYHEQPTFGSSLKWSSEGKSAFPVTIPRCIRPGYYLLRAEHIALHSASTPGGAQFYISCAQLAVTGGGSTDPPNKVSFPGAYKANDPGIQINIYYPVPTSYKNPGPSVFSC |
| *Chaetomium thermophilum* | EGS22639.1 | MKLTLTAALATIAAHEVAGHALFQQLWVDGTDYGASCIRMPMSNSPITNVNSRDFVCNAGTRPVSGKCPVRAGGTVTVEMHQQPGDRSCNNEAIGGAHWGPVQVYLSKVPDATTADGASTGWFKIFSNSWSKKPGSRSGDDDNWGTRDLNACCGKMDVKIPADLQDGDYLLRAEALALHTAGQAGGAQFYISCYQITVSGGTGTASPSLVRFPGAYGANDPGIMINIHSALDNYIAPGPPVYAGGTTKVAGSGCAGCESTCKVGSSPSAQAPTFEPVGGGTAGVPEASSCQVQPYGQCGGQGYTGCTQCASGYTCRAISPPYYSQCVAGA |
| *Chaetomium thermophilum* | EGS22278.1 | MKLSTFLALLVSAAAEAHYVFPSIANTPDWQYVRQTTNFQSNAPVTDVNSDQIRCYERIPGQGAQGIYNVTAGSVLNYNARASISHPGPMAVYIAKVPDGQSARTWDGKGRVWAKIYQDYPSVSGNGLTWPSMGKTSIPVTIPRCLQNGEYLLRAEHIGLHSASSPGGAQFYISCAQISVSGGSGTWNPKNLVSFPGAYTATHPGIQINIYWPVPTSYTPPGPAVETC |
